# The *Plasmodium falciparum* pseudoprotease SERA5 regulates the kinetics and efficiency of malaria parasite egress from host erythrocytes

**DOI:** 10.1371/journal.ppat.1006453

**Published:** 2017-07-06

**Authors:** Christine R. Collins, Fiona Hackett, Jonathan Atid, Michele Ser Ying Tan, Michael J. Blackman

**Affiliations:** 1 Malaria Biochemistry Laboratory, The Francis Crick Institute, London, United Kingdom; 2 Department of Pathogen Molecular Biology, London School of Hygiene & Tropical Medicine, London, United Kingdom; Wellcome Trust Sanger Institute, UNITED KINGDOM

## Abstract

Egress of the malaria parasite *Plasmodium falciparum* from its host red blood cell is a rapid, highly regulated event that is essential for maintenance and completion of the parasite life cycle. Egress is protease-dependent and is temporally associated with extensive proteolytic modification of parasite proteins, including a family of papain-like proteins called SERA that are expressed in the parasite parasitophorous vacuole. Previous work has shown that the most abundant SERA, SERA5, plays an important but non-enzymatic role in asexual blood stages. SERA5 is extensively proteolytically processed by a parasite serine protease called SUB1 as well as an unidentified cysteine protease just prior to egress. However, neither the function of SERA5 nor the role of its processing is known. Here we show that conditional disruption of the *SERA5* gene, or of both the *SERA5* and related *SERA4* genes simultaneously, results in a dramatic egress and replication defect characterised by premature host cell rupture and the failure of daughter merozoites to efficiently disseminate, instead being transiently retained within residual bounding membranes. SERA5 is not required for poration (permeabilization) or vesiculation of the host cell membrane at egress, but the premature rupture phenotype requires the activity of a parasite or host cell cysteine protease. Complementation of SERA5 null parasites by ectopic expression of wild-type SERA5 reversed the egress defect, whereas expression of a SERA5 mutant refractory to processing failed to rescue the phenotype. Our findings implicate SERA5 as an important regulator of the kinetics and efficiency of egress and suggest that proteolytic modification is required for SERA5 function. In addition, our study reveals that efficient egress requires tight control of the timing of membrane rupture.

## Author summary

Malaria, a disease that kills hundreds of thousands of people each year, is caused by a single-celled parasite that grows in red blood cells of infected individuals. Following each round of parasite multiplication, the infected red cells are actively ruptured in a process called egress, releasing a new generation of parasites. Egress is essential for progression to clinical disease, but little is known about how it is controlled. In this work we set out to address the function in egress of a *Plasmodium falciparum* protein called SERA5, an abundant component of the vacuole in which the parasite grows. We show that parasites lacking SERA5 (or lacking both SERA5 and a closely-related protein called SERA4) undergo accelerated but defective egress in which the bounding vacuole and red cell membranes do not rupture properly. This impedes the escape and subsequent replication of the newly-developed parasites. We also show that modification of SERA5 by parasites proteases just prior to egress is important for SERA5 function. Our results show that SERA5 is a ‘negative regulator’ of egress, controlling the speed of the pathway that leads to disruption of the membranes surrounding the intracellular parasite. Our findings increase our understanding of the molecular mechanisms underlying malarial egress and show that efficient egress requires tight control of the timing of membrane rupture.

## Introduction

Malaria is caused by protozoan parasites of the genus *Plasmodium*. Sporozoites introduced into the human host by the mosquito vector migrate to the liver where the parasite replicates to produce merozoites. These are released into the bloodstream, initiating the asexual blood stage of the infection in which the parasite goes through multiple rounds of intraerythrocytic replication and host cell destruction, producing gradually increasing parasitaemia that eventually leads to clinical disease. Like many intracellular pathogens, the parasite replicates within an intracellular membrane-bound compartment called a parasitophorous vacuole (PV). Replication is by schizogony, in which formation of a multinucleated schizont occurs before a budding or segmentation process generates the individual daughter merozoites. In *Plasmodium falciparum*, the most widespread agent of fatal malaria, each intraerythrocytic replication cycle takes ~48 h, with production of 16 or more merozoites per schizont.

During schizont development, a number of soluble parasite proteins accumulate within the PV lumen. Amongst the best characterized of these is a set of proteins belonging to the serine rich antigen (SERA) family, so named due to the presence of 27 or more consecutive Ser residues within the first-recognized member of the family, variously called 111 K antigen, P126, P140, SERA or SERP I but now referred to as SERA5 in *P*. *falciparum* (PlasmodDB ID PF3D7_0207600). A unifying feature of the SERA proteins, first noticed in SERA5 by Higgins et al. [1] then confirmed by x-ray crystallographic determination of the *P*. *falciparum* SERA5 central domain by Hodder and colleagues [2], is their possession of a central domain homologous to papain-like cysteine peptidases (clan CA, family C1). *SERA* family members are found in all *Plasmodium* genomes examined [3], and whilst the number of genes varies depending on the species, in all cases they fall into two classes: those that encode a Cys residue at the position equivalent to the nucleophilic Cys25 of papain (Cys-type); and those that possess a Ser codon at this position (Ser-type). Gene disruption analysis of the 9 *P*. *falciparum SERA* genes suggested that only two, *SERA5* (Ser-type) and *SERA6* (Cys-type), are important in the haploid asexual blood stage parasites [4–6], implying crucial roles for SERA5 and SERA6 in this clinically relevant part of the parasite life cycle. Very recent work using conditional mutagenesis has confirmed that disruption of the *P*. *falciparum SERA6* gene is lethal [7].

Release (egress) of daughter merozoites from the infected erythrocyte has long been known to be sensitive to cysteine protease inhibitors, including the selective covalent modifier trans-epoxysuccinyl-L-leucylamido(4-guanidino)butane (E64) (e.g. [8]). The resemblance of the SERA proteins to cysteine proteases, together with their subcellular localisation in the PV in both blood stages [4, 9, 10] and liver stages [11], has spurred interest in the possibility of the SERA proteins playing a role in egress. In support of this, disruption of a SERA family member that is highly expressed in mosquito stages of the parasite life cycle produced a defect in release of sporozoites from oocysts, structures on the basal surface of the insect midgut in which sporozoite biogenesis occurs [12]. Additionally, SERA5, which is the most abundantly-expressed family member in *P*. *falciparum* blood stages, was shown in early studies to be subjected to extensive proteolytic processing that coincided temporally with and was dependent upon egress [13–16], suggesting a link between SERA5 function and egress. Subsequent work in *P*. *falciparum* has uncovered the mechanism underlying this proteolytic processing. Minutes before egress, activation of a parasite cGMP-dependent protein kinase called PKG leads to the discharge of specialised merozoite organelles called exonemes which contain a subtilisin-like serine protease called SUB1 [17–19]. Upon secretion into the PV lumen, SUB1 cleaves both SERA5 and SERA6 at 2 or 3 discrete positions, releasing their central papain-like domains [10, 17]. The available evidence suggests that SERA6 possesses proteolytic activity that is activated by this processing [10]. In contrast, SERA5 has a non-enzymatic role in the parasite, since mutations predicted to abolish catalytic activity (e.g. substitution with Ala of the putative nucleophilic Ser596) had no phenotypic effect, whilst similar approaches failed to obtain viable parasites possessing a disrupted *SERA5* gene [6]. Thus there is considerable evidence that SERA5 and SERA6 have important functions in the parasite. However, whether any member of the SERA family has a role in blood stage egress and what that role may be, is unresolved.

Here we have used a conditional genetic approach combined with selective pharmacological tools to provide the first experimental evidence that SERA5 regulates egress in asexual blood stages of *P*. *falciparum*. Remarkably, rather than acting as a mediator of egress as previously suspected, we show that SERA5 enhances egress efficiency by acting as a negative regulator of the kinetics of egress.

## Results

### Efficient conditional ablation of SERA5 expression using the DiCre system

**Fig 1:**
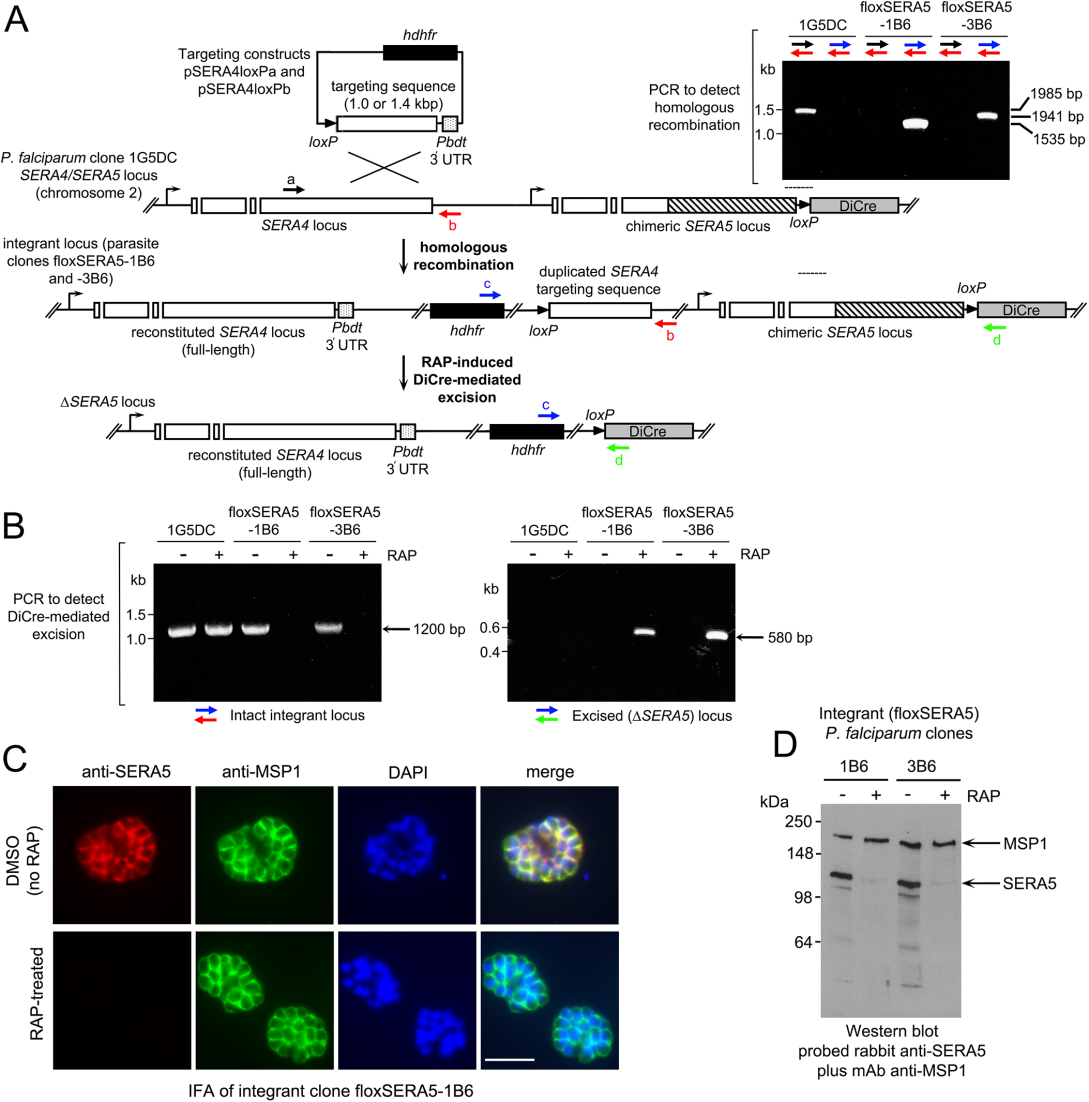
DiCre-mediated conditional disruption of *P*. *falciparum* SERA5 expression. (A) Strategy for conditional deletion of the *SERA5* gene. Transgenic *P*. *falciparum* clone 1G5DC contains a wild-type *SERA4* gene (white boxes, introns indicated as gaps) upstream of a fully functional chimeric *SERA5* gene (recodonised sequence, hatched). This is followed by a single *loxP* site (black arrow head) and the DiCre expression cassette (grey box). The targeting constructs contained ~1.0 kb (pSERA4loxPa) or ~1.4 kb (pSERA4loxPb) of 3´ *SERA4* sequence to drive integration of the entire construct by single-crossover homologous recombination. In both cases the targeting sequence extended to and included the *SERA4* stop codon and was followed by the 3′ UTR of the *P*. *berghei* dihydrofolate reductase (P*bdt*) gene to ensure correct regulation of the modified *SERA4* gene. Correct integration was expected to reconstitute the *SERA4* gene whilst introducing a second *loxP* site downstream of the introduced human dihydrofolate reductase-thymidylate synthase (*hdhfr*) selection cassette which confers resistance to the antifolate WR99210. DiCre-mediated recombination was predicted to excise the entire sequence between the *loxP* sites, including the *SERA5* gene. Positions of hybridisation of primers used for diagnostic PCR analysis of integration and excision events are shown as coloured arrows. Primer identities are: a, S4_F4; b, S4_DS_R1; c, CAM5´_R3; d, hsp86_3´_R1 (see S1 Table for sequences of primers used in this study). Insert, diagnostic PCR analysis of genomic DNA from the parental 1G5DC *P*. *falciparum* clone and integrant clones, confirming the predicted integration events. The expected sizes of the various PCR amplicons are indicated. (B) Diagnostic PCR analysis of genomic DNA from RAP-treated or control parental 1G5DC and integrant *P*. *falciparum* clones, confirming the predicted DiCre-mediated excision events. The expected sizes of the PCR amplicons specific for the intact or excised locus are indicated. (C) IFA of mature schizonts of control (DMSO-treated) and RAP-treated integrant clone floxSERA5-1B6 ~44 h following treatment. No SERA5-specific signal was detectable in the majority (~98%) of the RAP-treated population. Scale bar, 5 μm. (D) Western blot of mature schizonts of the integrant clones ~44 h following RAP-treatment or mock treatment, showing near complete loss of the SERA5 signal. As a loading control, blots were probed with antibodies to both SERA5 and the major merozoite surface protein MSP1. Quantitation of the residual SERA5 signal in the RAP-treated parasites (S1 Fig) indicated that this was likely due to lack of DiCre-mediated gene excision in a small proportion (~2%) of the parasite population.

In previous work [20] we described the production of a transgenic *P*. *falciparum* 3D7-derived clone called 1G5DiCre (here abbreviated to 1G5DC). These parasites possess a modified, partially recodonised (chimeric) *SERA5* gene encoding the wild type SERA5 amino acid sequence, followed by a single chromosomally-encoded *loxP* site and an integrated DiCre expression cassette. The latter drives constitutive expression of two individual, enzymatically inactive domains of Cre recombinase, each fused to a different rapamycin (RAP)-binding protein, such that addition of RAP induces heterodimerization of the proteins and Cre recombinase activity [21–23]. SERA5 protein expression by 1G5DC parasites is at wild type levels and the parasites replicate and egress normally in culture. Importantly, production of the 1G5DC clone included a step in which the human dihydrofolate reductase (*hdhfr*) drug resistance marker used to select for the desired homologous recombination event was removed from the genome by DiCre-mediated excision. The 1G5DC parasites are therefore fully sensitive to the antifolate drug WR99210 [20]. This recycling step allowed us to reuse the *hdhfr* selectable marker in a second gene targeting step (Fig 1A) in which a further *loxP* site was introduced into the parasite genome by targeted homologous recombination at the 3′ end of the upstream *SERA4* gene, reconstituting the *SERA4* gene whilst effectively floxing the entire chimeric *SERA5* locus. Two of the resulting transgenic parasite clones, called floxSERA5-1B6 and floxSERA5-3B6 were selected for further analysis. Examination by diagnostic PCR confirmed the expected genomic architecture (Fig 1A), whilst pulse-treatment of synchronised, newly-invaded (ring-stage) parasites with 100 nM RAP for just 1 h resulted in the expected DiCre-mediated excision event, rapidly deleting the entire chimeric *SERA5* coding sequence with high efficiency within a single erythrocytic cycle (Fig 1B).

To assess the effects of *SERA5* gene deletion on SERA5 protein expression, mature floxSERA5-1B6 and floxSERA5-3B6 schizonts were examined by indirect immunofluorescence (IFA) at the end of the first erythrocytic cycle (~44 h) following RAP-treatment. No SERA5-specific signal was detectable by IFA in most of the RAP-treated floxSERA5-1B6 and floxSERA5-3B6 schizonts (Fig 1C). Exhaustive microscopic examination showed apparently normal SERA5 expression in ~2% of schizonts (S1A Fig), likely representing a small population of parasites in which gene excision had not occurred. This was confirmed by Western blot analysis of schizonts of the RAP-treated floxSERA5-1B6 and floxSERA5-3B6 populations, indicating an overall >95% reduction of SERA5 expression (Fig 1D and S1B Fig). RAP-treatment had no detectable effect on expression of an unrelated merozoite protein, MSP1 (Fig 1C and 1D and S1B Fig), nor on expression of the *SERA4* and *SERA6* genes, which flank the *SERA5* gene (S1C Fig) [3, 5]. Schizont morphology and merozoite numbers in the ΔSERA5 parasites were normal by light microscopy of Giemsa-stained preparations (S1D Fig), and detailed visual examination of schizont SDS extracts fractionated on Coomassie-stained gels showed no detectable effects of RAP-treatment on the total parasite protein profile except for the noticeable absence of a ~120 kDa species identified by Western blot as full-length SERA5 (S1E Fig). These results were consistent with the genetic data, indicating specific, rapid and efficient DiCre-mediated disruption of SERA5 expression.

### SERA5 is important but dispensable for blood stage *P*. *falciparum* growth *in vitro*

**Fig 2:**
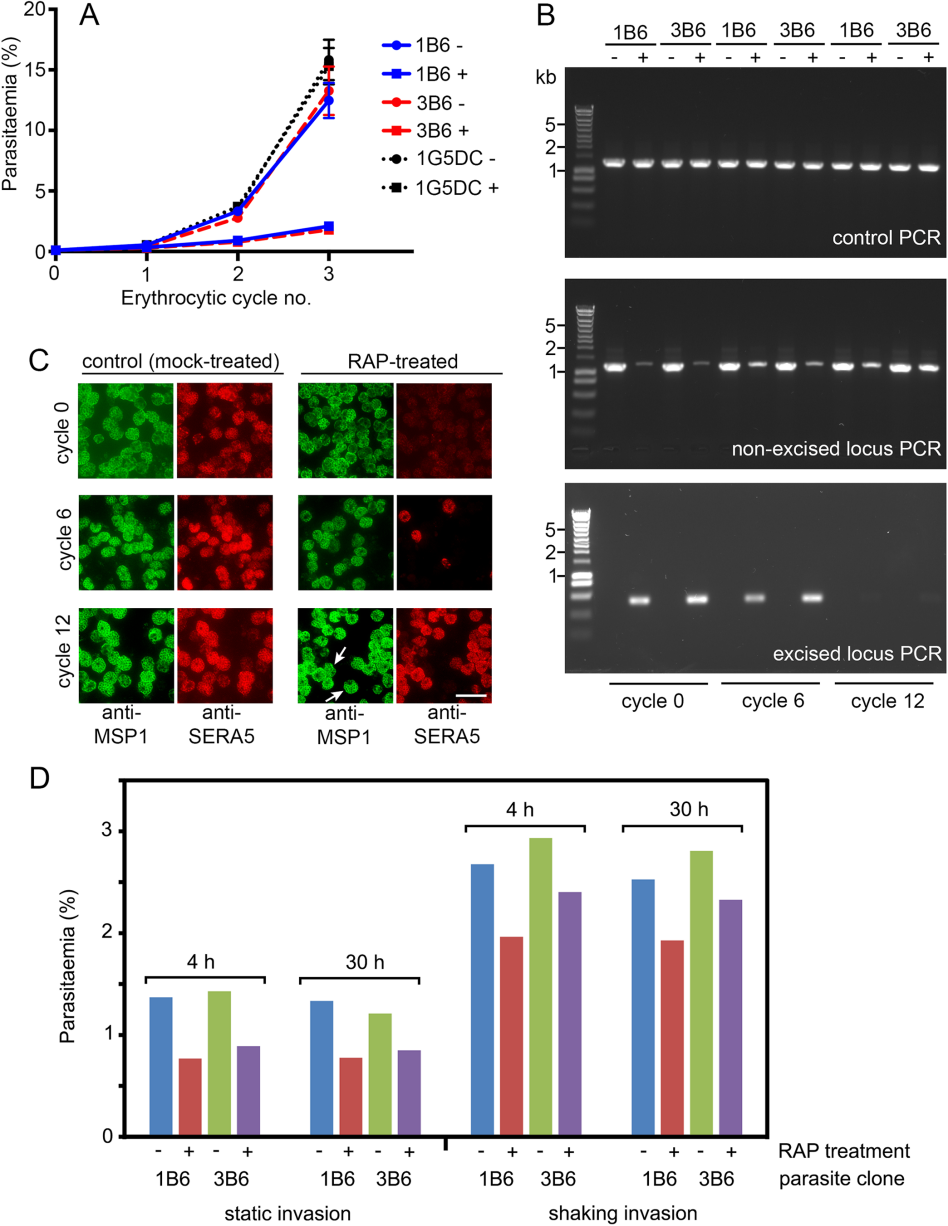
Loss of SERA5 expression results in severely reduced replication and invasion rates. (A) Relative replication rates of clones floxSERA5-1B6 and floxSERA5-3B6 compared to the parental 1G5DC clone, over the first 3 erythrocytic cycles following mock treatment (-) or RAP-treatment (+) at ring stage in cycle 0. Cultures were fed daily by replacing the medium, but were not passaged by addition of fresh erythrocytes. Parasitaemia values were determined by FACS at trophozoite stage as described in Materials and Methods. Data are compiled from 6 independent biological replicate experiments. Error bars, ±SEM. RAP-treatment of the floxSERA5-1B6 and floxSERA5-3B6 clones was calculated to result in a mean reduction in replication rate per cycle of 53±8%. (B) Diagnostic PCR analysis of genomic DNA isolated from parasites harvested at intervals following initiation of one of the assays depicted in (A) except that in this case the cultures were maintained for a more prolonged period (12 erythrocytic cycles), requiring regular passaging by dilution into medium containing fresh erythrocytes as described in Materials and Methods. Prolonged maintenance of the RAP-treated parasites resulted in selective expansion of the small number of non-excised parasites in the starting population, with concomitant substantial loss of parasites harbouring the excised locus (only just detectable by cycle 12). Primers used for amplification of the intact and excised loci were as in Fig 1, whilst primers specific for the *SERA4* locus were used for the control PCR. (C) IFA of purified schizonts, confirming outgrowth of SERA5-positive parasites in the RAP-treated floxSERA5-3B6 population, consistent with the PCR results. Whereas at cycle 0 (~44 h following RAP-treatment) the majority of the RAP-treated population were SERA5-negative, by cycle 12 very few ΔSERA5 parasites were left in the RAP-treated population (arrowed). However, the continued presence of the ΔSERA5 parasites showed that they can continue to replicate for prolonged periods *in vitro*. Scale bar, 20 μm. (D) Loss of SERA5 reduces ring formation but has no effect on intracellular development. Typical invasion assay showing formation and development of new ring stage parasites following rupture of RAP-treated or mock-treated floxSERA5-1B6 and floxSERA5-3B6 schizonts. Schizonts (11% parasitaemia) were incubated with fresh erythrocytes in static or shaking cultures for just 4 h before removal of residual intact schizonts by centrifugation over Percoll cushions. Parasitaemia was then determined by FACS following further incubation of the ring cultures in static conditions (early schizont stage; 30 h). RAP treatment produced a consistent reduction in ring formation, but had no or little impact on subsequent intracellular development. Similar results were obtained in three independent experiments.

To establish the effects of SERA5 loss on parasite growth, we compared the replication rates of mock-treated and RAP-treated floxSERA5-1B6 and floxSERA5-3B6 clones, using the parental 1G5DC clone as a control. No morphological or growth differences were evident in the ~44 h immediately following RAP-treatment (referred to as cycle 0), and all the parasites matured to schizont stage at the same rate (Fig 1C, S1A and S1D Fig) with no differences in the number of merozoites produced per schizont (S1D Fig; also see below). However, a ~50% reduction in the number of intracellular parasites was evident in the RAP-treated floxSERA5-1B6 and floxSERA5-3B6 cultures by the middle of the next cycle, and further monitoring into cycle 2 revealed a clear replication defect in these cultures (Fig 2A). Over a more prolonged period, periodic examination of the RAP-treated floxSERA5-1B6 and floxSERA5-3B6 cultures by IFA and diagnostic PCR indicated a time-dependent increase in the proportion of SERA5-expressing parasites in the cultures (Fig 2B and 2C), suggesting that the initially small population of non-excised parasites gradually overgrew the cultures, likely as a result of a selective advantage conferred on them by the replication defect displayed by the ΔSERA5 parasites. However, even after 12 erythrocytic growth cycles (24 days), ΔSERA5 parasites were still detectable in the RAP-treated cultures (Fig 2C, lower right-hand panels), proving that whilst loss of SERA5 expression severely impacted the rate of parasite replication, it did not completely abolish it.

To define the point(s) in the erythrocytic cycle affected by loss of SERA5 expression, parallel cultures of synchronous RAP-treated or mock-treated floxSERA5-1B6 or floxSERA5-3B6 mature schizonts at identical parasitaemia (11%) at the end of cycle 0 were allowed to undergo egress and invasion for just 4 h, under either normal static conditions or whilst continuously and vigorously shaken. Following removal of residual intact schizonts by centrifugation over Percoll cushions, the newly-formed ring-stage parasites were enumerated at once by FACS. Subsequent intracellular development (in static culture) of these parasites was also monitored by FACS approximately 30 h later, as well as by microscopic examination. As shown in Fig 2D, shaking the cultures during the invasion period increased ring formation in all cases, implying more efficient egress and/or invasion as previously observed by others [24], but production of rings was consistently reduced in the RAP-treated cultures compared to their mock-treated counterparts. However, once formed, these rings developed similarly over the course of the second intraerythrocytic cycle, irrespective of their provenance. Together with the previous evidence that SERA5-null parasites form morphologically normal schizonts, these results convincingly suggested that the reduction in long-term replication rate associated with loss of SERA5 was primarily or exclusively due to a defect not in intracellular parasite growth, but at the transition between schizont maturation and formation of new ring stage parasites.

**Fig 3:**
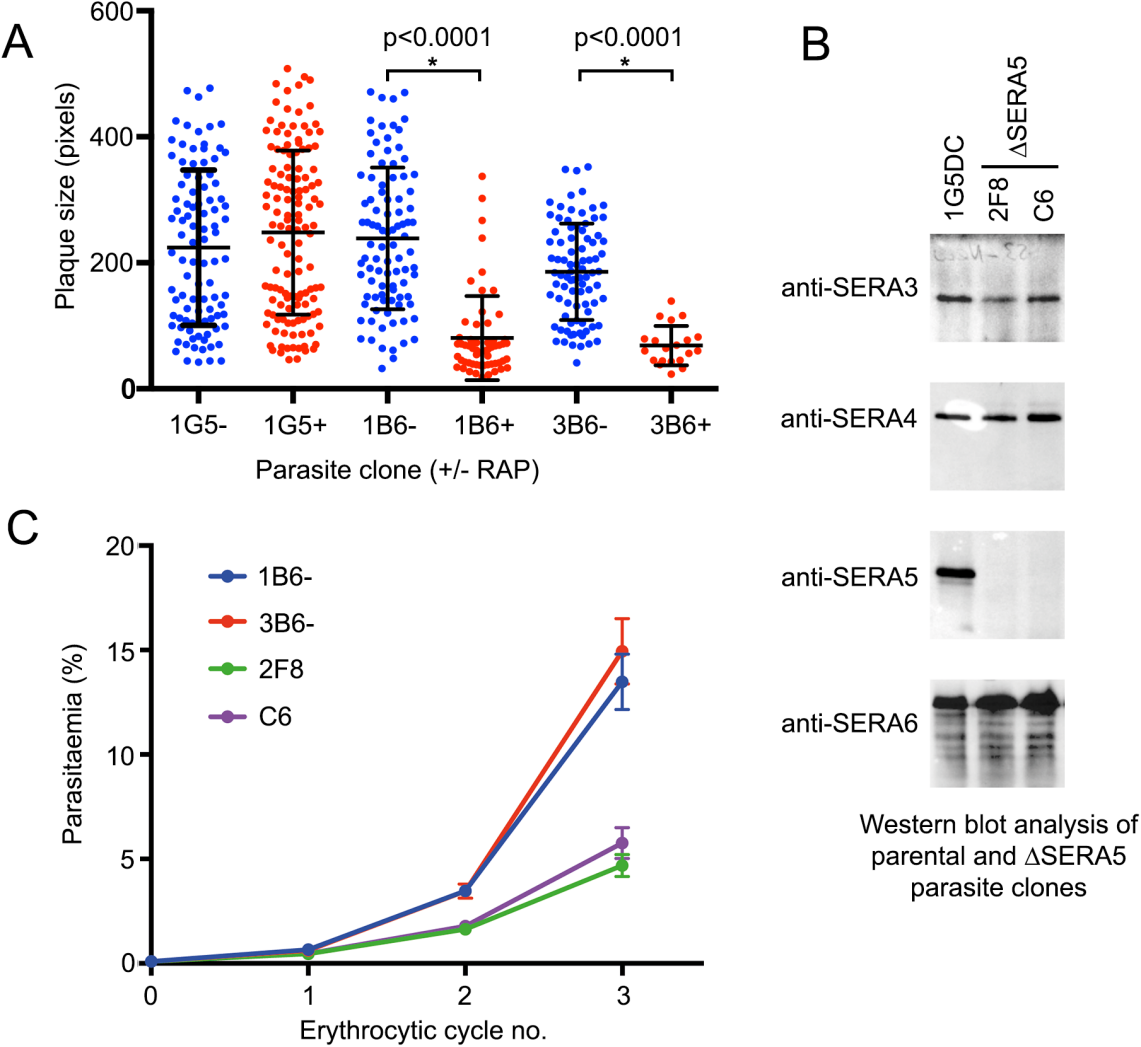
ΔSERA5 parasite clones show a replication defect but SERA5 is not essential for asexual blood stage growth *in vitro*. (A) Scatter plots showing plaque numbers and distribution of plaque sizes obtained following treatment of the parental 1G5DC clone or conditional knockout clones floxSERA5-1B6 and floxSERA5-3B6 with DMSO (control, mock-treated) or RAP to induce disruption of the *SERA5* gene. In each case plaque numbers were counted from a total of 24 microwells. Note that plaque numbers were lower in the RAP-treated floxSERA5-1B6 and floxSERA5-3B6 clones (n = 60 and 20 respectively) than in the mock-treated samples (n = 94 and 86 respectively). Plaque dimensions in scanned images of the plates were quantified using the Lasso tool of Photoshop CS6 (Adobe). Horizontal bars, mean plaque area ±1 SD. Brackets and asterisks indicate parasite populations for which statistically different plaque sizes were obtained (Student’s two-tailed *t*-test). The difference in plaque size for the +/- RAP-treated parental 1G5DC parasites were not significant (p-value = 0.1564). (B) Western blot confirmation of ΔSERA5 parasite clones obtained from plaque assay wells containing a single plaque. Clone 2F8_ΔSERA5 (derived from RAP-treated clone floxSERA5-1B6) and C6_ΔSERA5 (derived from RAP-treated clone floxSERA5-3B6) were expanded, then schizont extracts probed with antibodies to SERA3, SERA4, SERA5 and SERA6. (C) Growth assay showing relative replication rates of ΔSERA5 *P*. *falciparum* clones 2F8_ΔSERA5 and C6_ΔSERA5 compared to their parental floxSERA5-1B6 and floxSERA5-3B6 clones (not RAP-treated), over 3 erythrocytic cycles. Cultures were fed daily by replacing the medium, but were not passaged by addition of fresh erythrocytes. Parasitaemia values were determined by FACS as described in Materials and Methods. Data are averaged from 3 independent biological replicate experiments. Error bars, ±SEM. Relative to the parental parasites, the ΔSERA5 clones showed a mean reduction in replication rate per cycle of 28±3%.

To quantify the effects of SERA5 loss on long-term parasite growth in the absence of parasites still expressing SERA5, fresh floxSERA5-1B6 and floxSERA5-3B6 cultures were RAP- or mock-treated then identically diluted to obtain densities of <100 parasites/ml and transferred to flat-bottomed microwell plates. Examination of the plaques visible in these wells after 14–16 days (which form as a result of localised erythrocyte destruction in the static erythrocyte layers [7]) confirmed a growth defect in the ΔSERA5 parasites, with mock-treated floxSERA5-1B6 and floxSERA5-3B6 parasites producing significantly larger and more numerous plaques than their RAP-treated counterparts (Fig 3A). Parasites from 2 separate cloning wells of the RAP-treated floxSERA5-1B6 and floxSERA5-3B6 parasites, each of which contained only a single small plaque, were individually expanded. The resulting parasite clones (called 2F8_ΔSERA5 and C6_ΔSERA5) were confirmed as ΔSERA5 by Western blot (Fig 3B). They were then compared to the parental clones (not RAP-treated) in growth assays (Fig 3C). The results showed that loss of SERA5 results in a ~28% decrease in replication rate at each erythrocytic cycle. This difference in replication rates was rather less than the ~50% observed in static cultures immediately following *SERA5* gene excision (Fig 2A and 2D), suggesting the possibility of some adaptation of the 2F8_ΔSERA5 and C6_ΔSERA5 clones during the ~12 week period required for their cloning by limiting dilution and subsequent expansion. Nonetheless, it could be concluded unambiguously from these results that SERA5 is important for efficient blood stage parasite growth but is not essential for long-term viability *in vitro*.

### SERA5 null parasites undergo accelerated rupture but inefficient egress

**Fig 4:**
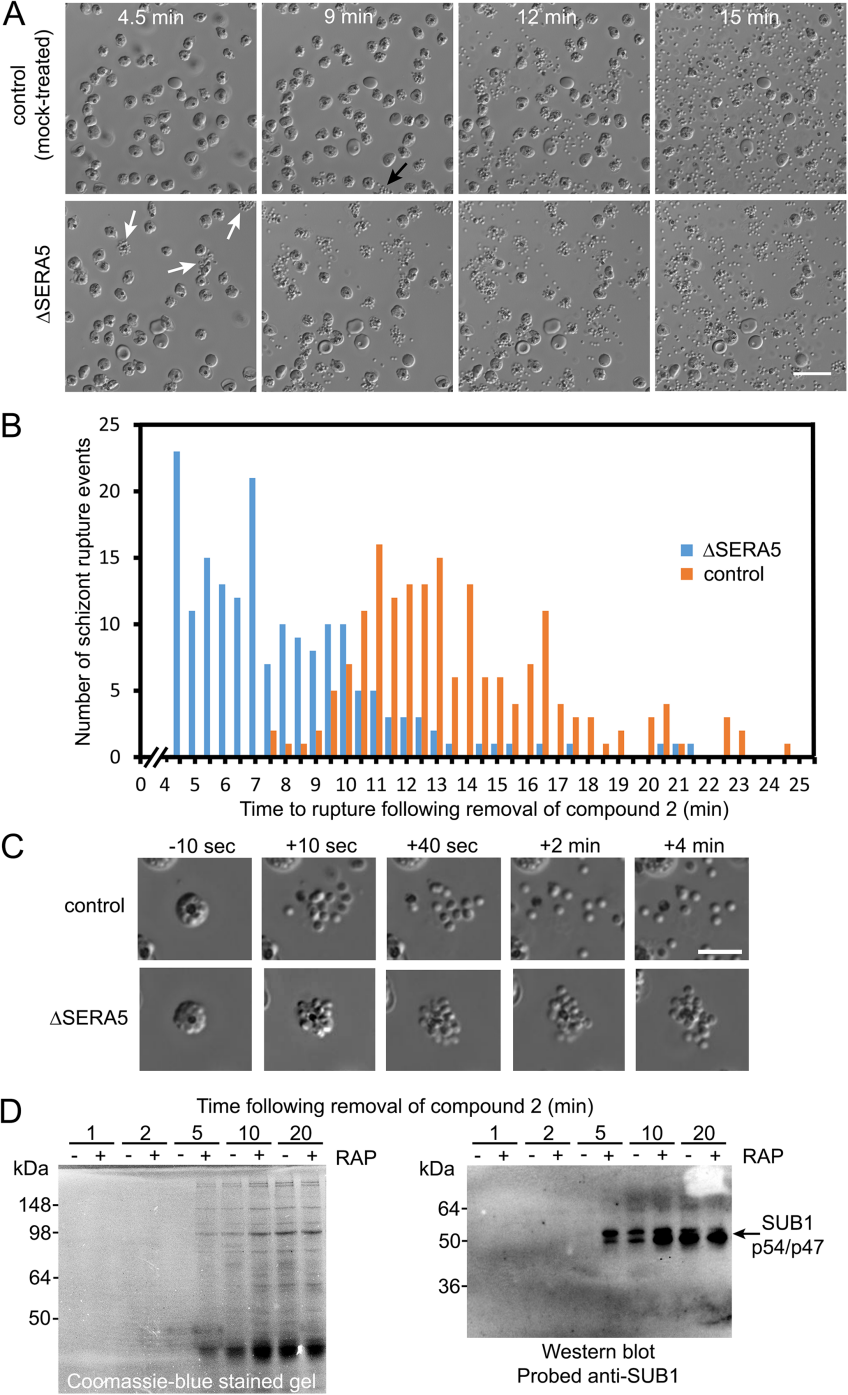
Loss of SERA5 results in accelerated schizont rupture but defective egress. (A) Stills from time-lapse DIC microscopic imaging showing stages in rupture of mock (DMSO)-treated (control) and RAP-treated (ΔSERA5) schizonts of *P*. *falciparum* clone floxSERA5-1B6. Movies were started exactly 4.5 min following removal of the reversible PKG inhibitor compound 2 (time following washing away the inhibitor is indicated). ΔSERA5 parasites underwent accelerated membrane rupture which had often already started even at the commencement of imaging (white arrows) with only gradual dispersal of the merozoites, unlike the ‘explosive’ egress and merozoite scattering typical of control parasites. Egress of the control parasites did not commence in this experiment until ~9 min (an example is indicated, black arrow). Scale bar, 20 μm. See also S1 Movie and S2 Fig. (B) Quantitative profiling of the timing of membrane rupture in the control and ΔSERA5 parasites. Whereas egress in the control parasites only rarely took place before 9 min, much of the membrane rupture evident in the ΔSERA5 parasites had already occurred by that point. Data were collated from visual examination of frames from 3 separate videos each of mock and RAP-treated clone floxSERA5-1B6 (total number of egress events: mock-treated, 178, RAP-treated, 179;). Time of egress is recorded to the nearest 0.5 min. Note that because imaging began 4.5 min following washing, all rupture events in the RAP-treated population that had already taken place at the beginning of the video microscopy are recorded as occurring at 4.5 min. Time to egress statistics were calculated for the RAP-treated parasites (mean 7.9 min, SD 3.2 min) and for the control parasites (mean 13.7 min, SD 3.3. min), with a two-tailed unpaired *t*-test revealing the difference to be extremely significant (t = 16.8268, d.f. = 355, p <0.0001). (C) Stills from time-lapse DIC microscopic images of individual control and ΔSERA5 schizonts, showing the very different characteristics of merozoite dispersal in the period immediately following the point of schizont rupture (the first still was imaged 10 seconds prior to rupture). Scale bar, 10 μm. (D) Coomassie-stained, SDS PAGE-fractionated and Western blot analysis of supernatants from mock and RAP-treated (ΔSERA5) floxSERA5-1B6 schizonts following release from a compound 2 block, showing more rapid release of schizont soluble proteins, including proteolytically processed forms of SUB1 [59] from the ΔSERA parasites. See also S3 Fig.

To gain insights into the defect underlying the reduced rates of new ring formation displayed by ΔSERA5 parasites, highly synchronous preparations of the floxSERA5-1B6 and floxSERA5-3B6 clones were RAP- or mock-treated at ring stage, then examined as they reached the point of egress. To enhance the synchrony of egress in these experiments, the purified mature schizonts (~43 h post-treatment) were transferred for 4–6 h into medium containing one or other of the PKG inhibitors 4-[2-(4-fluorophenyl)-5-(1-methylpiperidine-4-yl)-1H-pyrrol-3-yl] pyridine (compound 1), or (4-[7-[(dimethylamino)methyl]-2-(4-fluorphenyl)imidazo[1,2-*α*]pyridine-3-yl]pyrimidin-2-amine (compound 2) both of which potently but reversibly block parasite development at a stage just preceding egress [18, 25]. Short-term treatment of schizonts with either compound results in the accumulation of ‘stalled’ highly mature schizonts in which the PVM is intact though often porous [26]. Subsequent wash-out of the inhibitors releases the egress block, allowing the schizonts to proceed to rupture within minutes and facilitating time-lapse video microscopic examination of multiple egress events over a relatively short period [18, 26, 27]. As shown in Fig 4A–4C, as well as S1 Movie and S2 Fig, the RAP-treated floxSERA5-1B6 and floxSERA5-3B6 parasites displayed a dramatic egress phenotype characterized by abnormally rapid membrane rupture and inefficient dispersal of the released merozoites, which often initially formed asymmetric ‘clusters’ apparently due to them being transiently trapped within partially ruptured bounding membranes. The ΔSERA5 merozoites generally gradually dispersed from these clusters over a period of several minutes, proving that both the PV and host erythrocyte membranes had undergone rupture (Fig 4C and S2 Fig). Indeed, in many cases, PVM rupture clearly occurred shortly before erythrocyte membrane rupture in both mock-treated and ΔSERA5 parasites. This was indicated by transient rounding-up of the schizonts with swelling of the PV, then sudden loss of differential interference contrast of the cell and increased visibility and motility of the internal merozoites (S1 Movie), as previously observed in both *P*. *falciparum* [26, 28–30] and the zoonotic malarial species *P*. *knowlesi* [31]. The total proportion of schizonts that underwent rupture over the imaging periods did not differ between parallel mock and RAP-treated cultures, confirming that egress was not inhibited by loss of SERA5 expression. In both mock and RAP-treated schizonts, egress was often accompanied by sudden lateral displacement of the schizont by as much as 5–10 μm, perhaps indicating rapid expulsion of the cytosolic contents of the cell upon the release of the osmotic pressure proposed by some to be responsible for the pre-egress PV swelling [28, 32]. The time interval between washing away the PKG inhibitors and initiation of egress in control (mock-treated) parasites differed somewhat between the PKG inhibitors, with a rather longer delay in the case of compound 1 (minimum delay to egress ~13 mins) compared to compound 2 (minimum time to egress ~7.5 min). However, in all cases the ΔSERA5 parasites exhibited significantly accelerated membrane rupture compared to similarly treated controls. This was confirmed by Coomassie-staining and Western blot analysis, which showed more rapid appearance of soluble parasite-derived proteins in the supernatants of RAP-treated floxSERA5-1B6 schizonts released from a compound 2-mediated egress block than in the mock-treated samples (Fig 4D). In contrast, Western blot analysis of the schizonts themselves showed no discernible difference in the rates of proteolytic processing of two established SUB1 substrates, SERA6 and MSP1 (S3 Fig). Importantly, the abortive egress phenotype and formation of merozoite ‘clusters’ was also observed in mature ΔSERA5 schizonts that had not been synchronised by treatment with compounds 1 or 2, confirming that the phenotype was not an artefact associated with prior exposure to the PKG inhibitors (S2 Movie). Time-lapse microscopic examination of the 2F8_ΔSERA5 and C6_ΔSERA5 clones compared with RAP-treated parental 1G5DC parasites (S3–S5 Movies) confirmed the premature egress phenotype in the ΔSERA5 clones and showed that these parasites had not undergone major compensatory phenotypic alterations during the extended culture period (~12 weeks) required for their isolation by limiting dilution cloning and expansion. This phenotype was still evident even after extended continuous culture (>16 months) of these parasite clones. It was concluded that loss of SERA5 results in accelerated schizont rupture but defective egress, and that this likely explains the replication defect evident in the ΔSERA5 parasites.

### SERA5 null parasites are not intrinsically defective in host erythrocyte membrane poration or vesiculation

**Fig 5:**
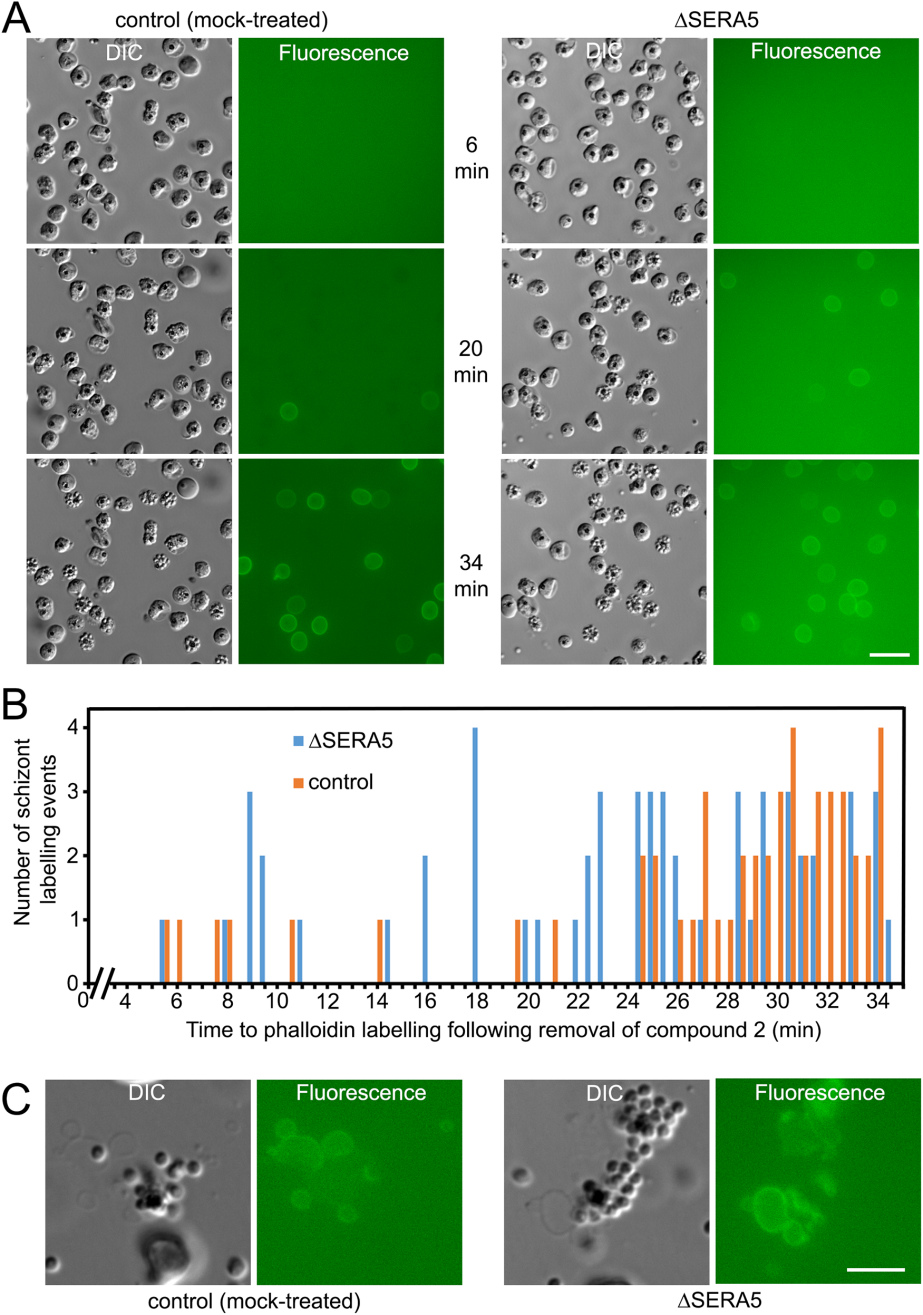
SERA5 does not play a role in host cell membrane poration or vesiculation at egress. (A) Stills from simultaneous time-lapse DIC and fluorescence microscopic imaging showing ingress of Alex Fluor 488-labelled phalloidin into intact mock-treated (control) and RAP-treated (ΔSERA5) schizonts of *P*. *falciparum* clone floxSERA5-3B6 in the presence of E64. Movies were started exactly 4.5 min following removal of compound 2 (time following washing away the inhibitor is indicated). Scale bar, 20 μm. (B) Quantitative profiling of the timing of phalloidin labelling in the control and ΔSERA5 schizonts. Data were collated from visual examination of frames from 3 separate videos each of mock and RAP-treated parasites (total number of phalloidin labelling events: RAP-treated, 56; mock-treated, 51). Time to labelling is indicated to the nearest 0.5 min. Statistics were calculated for the ΔSERA5 parasites (mean 23.7 min, SD 7.9 min) and the control parasites (mean 27.2 min, SD 7.6. min), with a two-tailed unpaired *t*-test revealing the difference to be just significant (t = 2.3260, d.f. = 105, p = 0.0219). (C) Stills from simultaneous time-lapse DIC and fluorescence microscopic imaging of control and ΔSERA5 floxSERA5-3B6 schizonts in the presence of fluorescent phalloidin but the absence of E64, showing vesiculation and phalloidin labelling of the disrupted host cell membranes following egress. Note that the released merozoites are slightly out of focus in these images in order to focus on the vesiculated membranes. Scale bar, 10 μm. See also S4 Fig.

Elegant previous studies by Glushakova et al. and others in *P*. *falciparum* have demonstrated that, in the seconds just preceding host red blood cell membrane breakage at egress, the membrane becomes permeabilized or ‘porated’, allowing leakage of cellular contents and the ingress of extracellular molecules [26, 32, 33]. The latter includes the F-actin-binding peptide phalloidin which, if introduced into the surrounding culture medium, is able just before egress to access and label the actin protofilaments of the cytoskeleton that underlies the host erythrocyte cell membrane. By allowing schizont rupture in the presence of fluorescent phalloidin, poration can be detected by microscopy as the sudden appearance of a fluorescent signal around the host cell circumference [32, 33]. In addition, phalloidin binding to the ruptured host erythrocyte cytoskeleton immediately after egress enables visualisation of the state of the fragmented membranes, which generally undergo extensive vesiculation [28, 32]. Host erythrocyte membrane rupture (but not PVM rupture) is selectively blocked by the cysteine protease inhibitor E64 but poration of the membrane is not [26, 32], so the combined use of E64 and fluorescent phalloidin provides a simple means of reliably observing the onset and efficiency of poration, since the porated schizonts do not rupture. To examine whether loss of SERA5 expression impacts on poration and fragmentation of the erythrocyte membrane, compound 2-synchronised, mock or RAP-treated floxSERA5-3B6 schizonts were washed and transferred to fresh medium containing Alexa Fluor 488-labeled phalloidin, with or without E64. Simultaneous time-lapse DIC and fluorescence imaging of the E64-containing cultures showed the gradual appearance of phalloidin-labelled schizonts as the host cell membrane of these cells became porated (Fig 5A). Intriguingly, the appearance of phalloidin labelling in individual schizonts was always temporally coincident with PVM rupture, the latter being clearly evident by DIC imaging as the sudden loss of differential interference contrast of the schizont and increased visibility and mobility of the intracellular merozoites (S6 Movie). Of additional interest, compared to the kinetics of egress in parallel samples lacking E64 which progressed rapidly to rupture with kinetics as described in Fig 4, phalloidin labelling in the presence of E64 was substantially delayed in both control and ΔSERA5 samples (compare Fig 5B with Fig 4B). This is the first evidence to our knowledge that although E64 does not prevent PVM rupture, it delays the process. Whilst the rate of appearance of phalloidin-labelled schizonts in the presence of E64 was slightly faster in the ΔSERA5 schizont population (Fig 5A and 5B), this was only just significant, in contrast to the clear premature rupture phenotype displayed by the ΔSERA5 parasites in the absence of E64. The total proportion of schizonts labelled with phalloidin within a ~35 min period following removal of the compound 2 block in the presence of E64 was similar for the ΔSERA5 and control cultures (Fig 5B). Time-lapse DIC examination of E64-containing cultures in the absence of phalloidin detected no significant difference between the timing of PV rupture in the control and ΔSERA5 populations (S4 Fig). In cultures containing phalloidin but lacking E64, extensive vesiculation of the residual host cell membranes was observed in all cases following egress (Fig 5C). Collectively, these results showed that SERA5 does not play a direct role in mediating host cell membrane poration or vesiculation. However, the fact that in the presence of E64 the rate of progress to PVM rupture and host cell membrane poration in the ΔSERA5 parasites was very similar to that of control parasites, implied a potential link between SERA5 function and the protein target(s) of E64. This point is further discussed below.

### Simultaneous disruption of both the *SERA4* and *SERA5* genes produces an egress phenotype indistinguishable from that resulting from *SERA5* disruption

**Fig 6:**
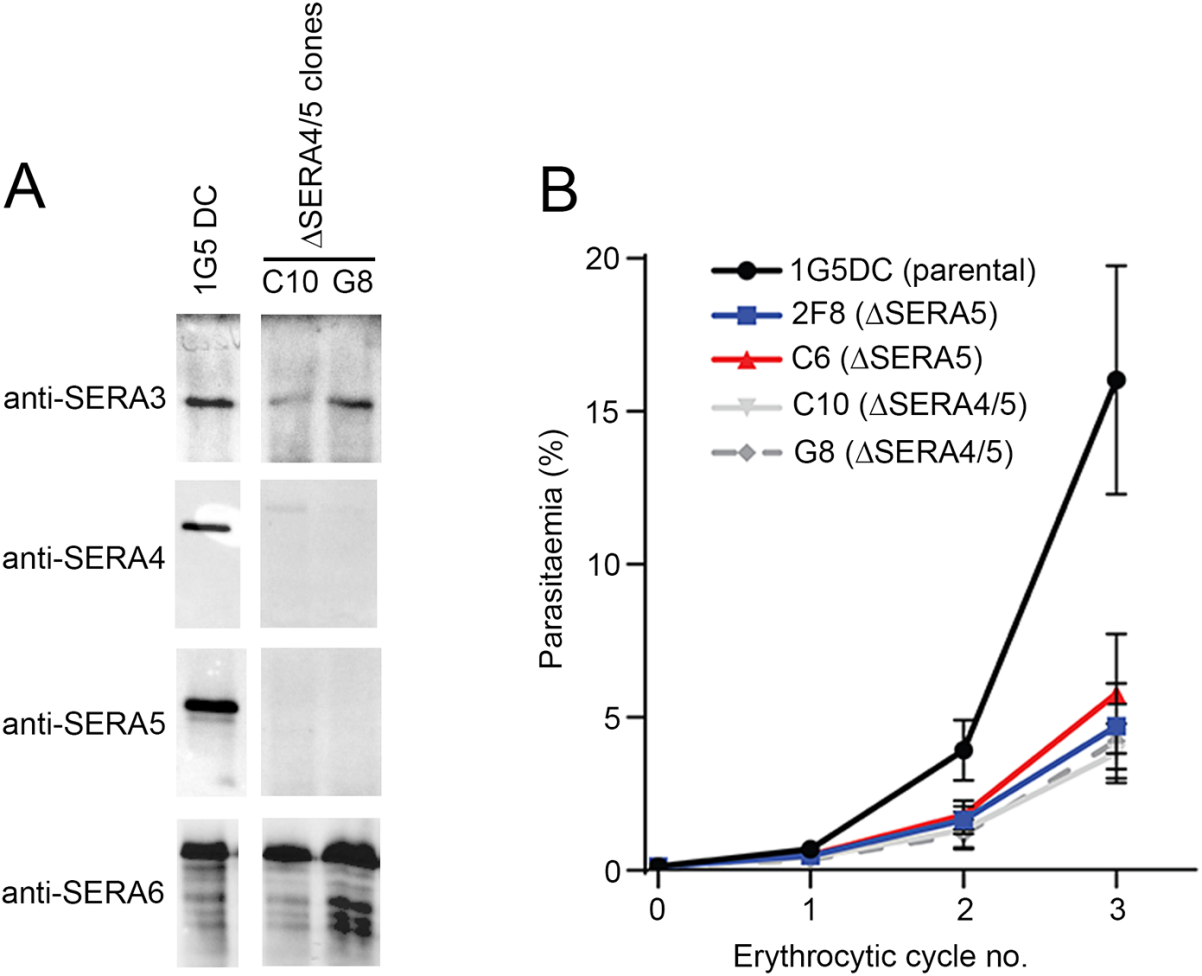
Simultaneous disruption of both the *SERA4* and *SERA5* genes results in a replication defect similar to that produced by *SERA5* disruption alone. (A) Western blot analysis of the C10_ΔSERA4/5 and G8_ΔSERA4/5 *P*. *falciparum* clones (as well as the 1G5DC parental clone as a control), confirming loss of expression of both SERA4 and SERA5 in the double knockout parasites (note that the 1G5DC loading control blots are the same as shown in Fig 3, since all the Western blots were performed at the same time). (B) Growth assay showing relative replication rates of the ΔSERA4/5 *P*. *falciparum* clones compared to the single knockout 2F8_ΔSERA5 and C6_ΔSERA5 clones, over 3 erythrocytic cycles. Cultures were fed daily by replacing the medium, but were not passaged by addition of fresh erythrocytes. Parasitaemia values were determined by FACS. Data are averaged from 5 independent biological replicate experiments. Error bars, ±SEM. See also S5 Fig.

The genes encoding the Ser-type *SERA* family members *SERA1-5* in *P*. *falciparum* lie in a head-to tail tandem array on chromosome 2 [3, 5]. This, together with the high degree of synteny between *SERA* loci in all *Plasmodium* species examined, suggests that the Ser-type *SERA* genes arose through gene duplication events, the number of which varied between different *Plasmodium* species [3]. Phylogenetic analyses have suggested that all the Ser-type SERA proteins may mediate similar functions [3, 5]. However, expression levels vary widely, with both transcriptional and proteomic data indicating that in *P*. *falciparum* SERA5 is the most highly expressed, whilst SERA4 is the second most abundantly expressed [4, 34, 35] and is dispensable in blood stages [5]. In the light of our success in flanking the *SERA5* gene on the 1G5DC background with *loxP* sites using a single-crossover homologous recombination approach, we decided to employ a modified strategy to simultaneously flox both the *SERA5* and *SERA4* genes with the aim of examining the consequences of disrupting both genes simultaneously. As shown in S5 Fig, a construct (pSERA3loxP) designed to insert a *loxP* site immediately downstream of the *SERA3* gene successfully integrated in the expected manner into the 1G5DC genome. Treatment with RAP of a parasite clone (called floxSERA4/5-B52) harbouring the integrated construct resulted in excision of both the *SERA4* and *SERA5* genes as shown by diagnostic PCR analysis (S5 Fig). The resulting parasites were cloned by limiting dilution and two clones obtained. Examination by Western blot of mature schizonts of these clones (called C10_ΔSERA4/5 and G8_ΔSERA4/5) confirmed the absence of both SERA4 and SERA5 but unaltered expression of SERA3 and SERA6, as expected (Fig 6A).

To assess the effects of simultaneous loss of both SERA4 and SERA5 on parasite egress, we examined the timing and morphology of egress of the floxSERA4/5-B52 parasites following mock-treatment or treatment with RAP. As shown in S7 Movie and S8 Movie, the ΔSERA4/5 parasites displayed an accelerated but defective egress phenotype identical to that of ΔSERA5 parasites. Growth assays comparing the replication rates of the ΔSERA4/5 clones with the ΔSERA5 clones showed no significant differences in replication rates over the course of 3 erythrocytic cycles (Fig 6B). It was concluded that simultaneous loss of both SERA4 and SERA5 produces a defect in egress no more severe than that resulting from loss of SERA5 alone.

### Episomal expression of the wild-type *SERA5* gene, but not a mutant refractory to SUB1-mediated cleavage, rescues the growth defect associated with *SERA5* knockout

**Fig 7:**
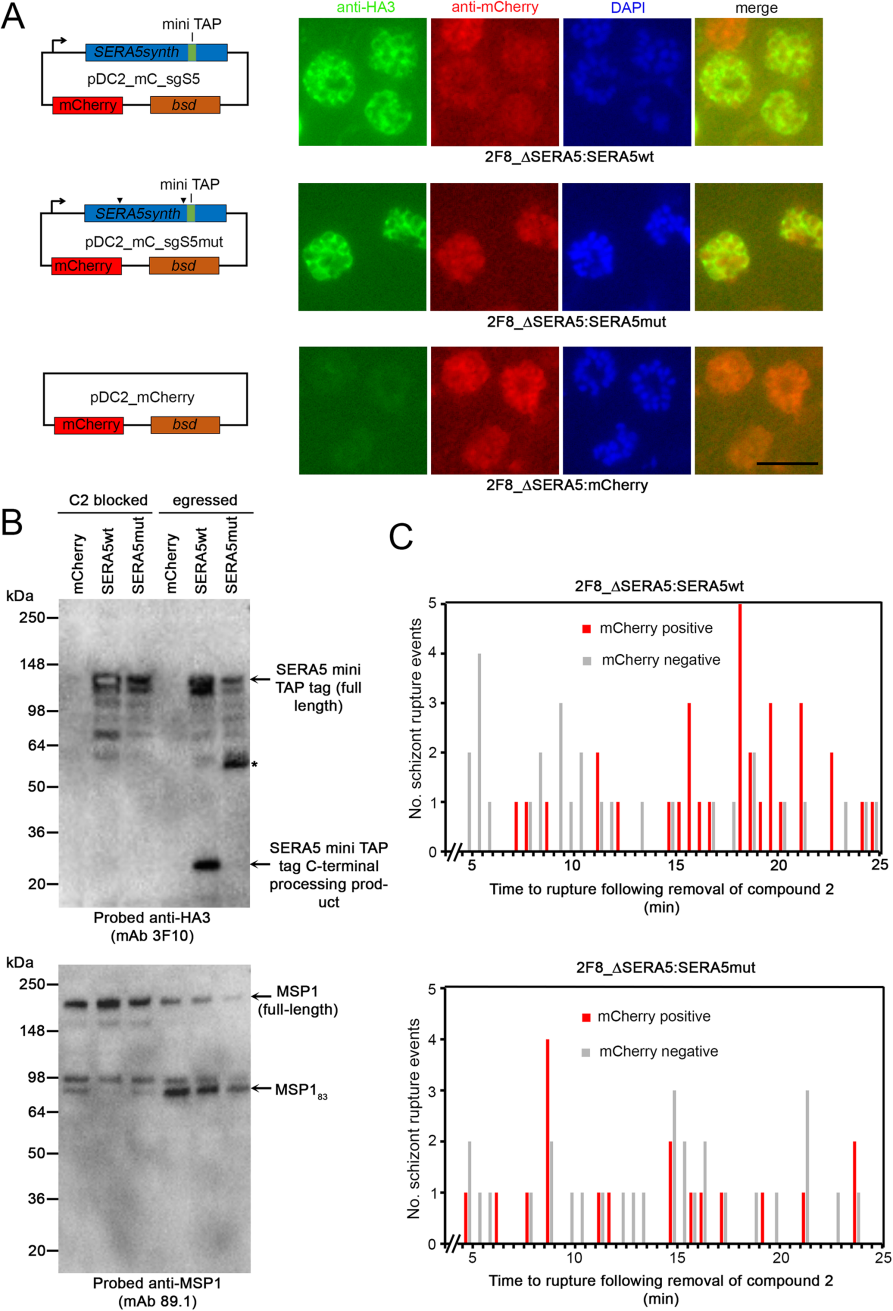
Genetic complementation experiments show that proteolytic processing of SERA5 is important for its function. (A) Left-hand side: transgene constructs for genetic complementation of the ΔSERA5 parasite clone 2F8_ΔSERA5. All constructs were designed for constitutive expression of cytoplasmic mCherry, driven by the *P*. *falciparum* CAM promoter. Constructs pDC2_mC_sgS5 and pDC2_mC_sgS5mut additionally incorporated cassettes for expression of a synthetic recodonised *SERA5* gene (under control of the *SERA5* promoter) modified by inclusion of an internal mini TAP tag which includes the HA3 epitope [6]. Positions of mutations (see S6 Fig for details of mutations) introduced into pDC2_mC_sgS5mut to prevent processing by SUB1 and protease X [6] are indicated (arrowheads). *bsd*, blasticidin deaminase drug resistance cassette. Right-hand side: IFA of the blasticidin-resistant parasite lines selected following transfection of the SERA5-null parasite clone 2F8_ΔSERA5 with the constructs. Reactivity with the anti-HA3 mAb 3F10 was observed only in mature schizonts of the 2F8_ΔSERA5:SERA5wt and 2F8_ΔSERA5:SERA5mut lines, in a pattern typical of PV localisation. For clarity, the merged images do not include the DAPI signal. Scale bar, 10 μm. (B) Western blot analysis of purified mature schizonts of the transfected parasite lines, either following incubation for 4 h in the presence of the PKG inhibitor compound 2 (C2 blocked) or 40 min after washing away of compound 2 to allow egress of the majority of the schizonts (egressed). This involves discharge of SUB1 and proteolytic processing of SERA5 and the merozoite surface protein MSP1 [18, 27]. HA3-tagged SERA5 was expressed only in the 2F8_ΔSERA5:SERA5wt and 2F8_ΔSERA5:SERA5mut lines. As expected, proteolytic processing of the protein was defective in the 2F8_ΔSERA5:SERA5mut line, leading to a species (asterisked) that was much larger than the HA3-tagged processing product of wild-type SERA5 (indicated and labelled). In contrast correct SUB1-mediated processing of MSP1 to form the MSP1_83_ processing product [27] occurred similarly in all three parasite lines. (C) Quantitative profiling plot of the timing of egress in the 2F8_ΔSERA5:SERA5wt and 2F8_ΔSERA5:SERA5mut parasite lines. In each case, time to egress of mCherry-positive schizonts (red) following washing away of a compound 2 block is shown compared with that of mCherry-negative parasites (grey) in the same fields. The mCherry-positive schizonts were defined as those with a mean fluorescence intensity value of ≥30 under standardised fluorescence imaging conditions (see Materials and Methods). Time-lapse imaging began precisely 4.5 min following washing, and data were collated from visual examination of frames from 3 separate videos each of the two lines. Time to egress statistics were calculated for the mCherry-positive 2F8_ΔSERA5:SERA5wt parasites (mean 17.08 min, SD 4.52 min) and for the mCherry-negative 2F8_ΔSERA5:SERA5wt parasites (mean 12.0 min, SD 6.42 min), with a two-tailed unpaired *t*-test revealing the difference to be extremely significant (t = 3.5985, d.f. = 59, p <0.001). In contrast, similar analysis of the time to egress statistics for the mCherry-positive 2F8_ΔSERA5:SERA5mut parasites (mean 13.28 min, SD 5.91 min) and mCherry-negative 2F8_ΔSERA5:SERA5mut parasites (13.69 min, SD 5.57 min) revealed the difference to be non-significant (t = 0.2397, d.f. = 45, p = 0.812). See also S9 Movie and S10 Movie.

The very similar phenotype displayed by the ΔSERA5 and the ΔSERA4/5 parasites, together with the conditional nature of the gene disruption strategy and the apparently unaltered expression of the flanking *SERA3* and *SERA6* genes in both sets of knockouts, gave us a high degree of confidence that the observed phenotype was a direct result of manipulation of the *SERA4/5* loci and not due to unintended genetic alterations in the modified parasites. To confirm this and to investigate the structural requirements for SERA5 function, we sought to rescue the ΔSERA5 phenotype by genetic complementation. For this, we introduced into 2F8_ΔSERA5 parasites a transgene construct (called pDC2_mC_sgS5) designed for episomal expression of the *SERA5* gene under the control of endogenous flanking 5’ sequence likely to include the genomic *SERA5* promoter (Fig 7A). To reliably identify the transgene product, the *SERA5* transgene was modified by the introduction of an internal mini TAP tag sequence incorporating a hemagglutinin (HA3) epitope tag, that we had previously shown does not interfere with SERA5 function in the parasite, despite abolishing the SUB1 site 2 processing site [6]. The pDC2_mC_sgS5 construct also contained a cassette for constitutive expression of cytoplasmic mCherry to facilitate identification of parasites harbouring the construct. In parallel, 2F8_ΔSERA5 parasites were independently transfected with a mutant of the same episome, called pDC2_mC_sgS5mut, in which the *SERA5* sequence encoding the site 1 SUB1 processing site [17], as well as a site that is cleaved by an anonymous cysteine protease referred to as protease X [6], were modified in a manner designed to block correct cleavage of the transgene product (Fig 7A). A third set of 2F8_ΔSERA5 parasites were transfected with a similar construct that contained the mCherry expression cassette but lacked the SERA5 expression cassette. Transfected parasites were subjected to selection in the presence of blasticidin. IFA and Western blot analysis of the resulting three blasticidin-resistant lines (called 2F8_ΔSERA5:SERA5wt, 2F8_ΔSERA5:SERA5mut and 2F8_ΔSERA5:mCherry) showed mCherry expression in all cases (although not in every parasite; see below) and confirmed expression of the transgenic SERA5 proteins in the 2F8_ΔSERA5:SERA5wt and 2F8_ΔSERA5:SERA5mut lines (Fig 7A and 7B), as well as the expected SERA5 processing defect in the mutant line (Fig 7B). To examine the effects of transgene expression on the egress phenotype, egress of synchronous mature schizonts of the 2F8_ΔSERA5:SERA5wt and 2F8_ΔSERA5:SERA5mut parasite lines following release of a compound 2-mediated block was visualised by simultaneous live DIC and fluorescence time-lapse imaging as previously. Substantial variation in the levels of mCherry expression was evident in the imaged schizonts, likely due to heterogeneity in episome segregation as previously noted for episomal plasmids in *P*. *falciparum* [36, 37]. However comparison of the fluorescent with the non-fluorescent schizonts in each population clearly showed rescue of the delayed (Fig 7C) and abortive (S9 Movie and S10 Movie) egress phenotype in the 2F8_ΔSERA5:SERA5wt parasites but not in the mutant line. It was concluded that correct proteolytic processing of SERA5 is likely essential for its function in regulating the kinetics of egress.

## Discussion

Like all obligate intracellular pathogens, the malaria parasite has evolved efficient molecular strategies to exit from its host cell. In this study we have made inroads into our understanding of the regulatory mechanisms underlying blood stage egress in *P*. *falciparum*, showing that conditional disruption of the *SERA5* gene, or both the *SERA4* and *SERA5* genes simultaneously, leads to a dramatic egress defect characterised by premature but inefficient membrane rupture. The released daughter merozoites disseminate slowly, apparently due to their being retained transiently within residual, incompletely fragmented bounding membranes. The result is a reduction in invasion of new host erythrocytes. The indistinguishable phenotypes of the ΔSERA5 and ΔSERA4/5 mutants is consistent with the notion of functional redundancy amongst the Ser-type *SERA* genes and previous evidence that loss of SERA4 alone is tolerated by the parasite [5].

**Fig 8:**
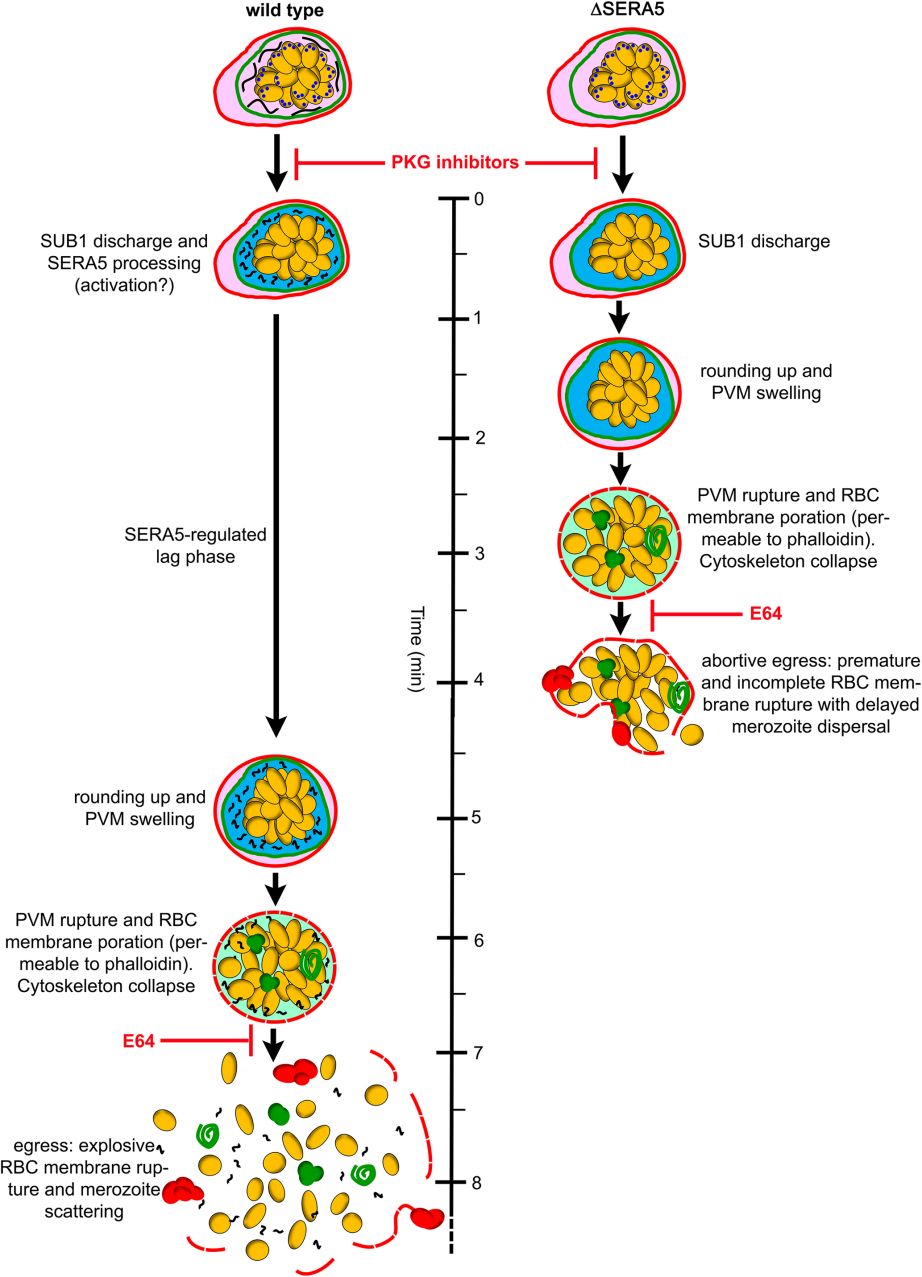
Model for role of SERA5 in regulating a lag phase that enables efficient bounding membrane disruption at egress. Schematic model of the role of SERA5 based on the results of this and previous studies. The erythrocyte and PV membranes of mature schizonts are indicated in red and green respectively. SUB1 in exonemes of mature segmented schizonts is depicted as blue dots, whilst full-length SERA5 is shown as black strands within the PV. Evidence that the PVM becomes porous prior to PKG activation, that PVM disruption results in vesiculated ‘rolls’ of membrane and that erythrocyte membrane rounding up and/or poration is associated with rapid collapse of the host cell cytoskeleton is from earlier work [26, 32]. Timelines shown to egress are approximate minima and are based on reversible inhibition of egress by compound 2. The final step of host cell membrane rupture is sensitive to E64 in both wild type and ΔSERA5 parasites. However, in the presence of E64 the time to PVM rupture is similar for wild type and ΔSERA5 mutants (not indicated schematically). RBC, red blood cell.

Our study provides two important insights into the molecular mechanisms underlying blood-stage egress in *P*. *falciparum*. The first concerns the kinetics of the egress pathway downstream of PKG activation. Our work confirms our previous observations [18, 26] that there is a delay of several minutes between washing away the PKG inhibitors from compound 1 or compound 2-stalled schizonts, and initiation of egress. Here we quantified this period under the conditions used to a minimum of ~13 mins in the case of compound 1 and ~7.5 min for compound 2. We had previously assumed that this delay to egress is primarily a reflection of the rate of dissociation of the respective inhibitor-PKG complexes, and that egress initiates as soon as release of the bound drug allows reactivation of sufficient PKG activity to trigger egress. However our new data force a re-evaluation of that simplistic assumption, because schizont membrane rupture consistently occurs more rapidly in the ΔSERA5 and ΔSERA4/5 mutants. The premature rupture is clearly PKG-dependent, since it is reversibly blocked by PKG inhibitors. It also involves SUB1 discharge (Fig 4C), so our observations reveal for the first time that the delay to egress observed in wild-type parasites following removal of the PKG inhibitors includes a ‘lag phase’ that is additional to the time required for the parasites to become membrane rupture-competent following PKG activation and SUB1 discharge into the PV (Fig 8). What is the function of this lag phase and how is it controlled?

The answers to these questions may lie in the second important insight provided by our study. The simplest interpretation of the premature rupture phenotype is that SERA5 (and also perhaps SERA4) is a negative regulator of the egress pathway, acting to maintain the lag phase and thus delay rupture of the PVM and host erythrocyte membranes following SUB1 discharge. The fact that the premature egress phenotype is also associated with a defect in merozoite dissemination suggests that the function of the lag phase is to ensure proper destabilisation of the bounding membranes and/or collapse of the host cell cytoskeleton [26] such that, once they rupture, the merozoites can easily and rapidly escape from the residual membranes. Elegant high speed video microscopy and modelling experiments by Abkarian and colleagues [30] has shown that the scattering of merozoites normally observed at schizont rupture is at least in part due to the elastic properties of the erythrocyte membrane which, upon primary rupture at a single site, undergoes extremely rapid curling and buckling, turning inside-out to literally fling the remaining enclosed merozoites outwards. This demonstrably does not happen in the ΔSERA5 and ΔSERA4/5 mutants. We suggest that in the absence of the SERA5-regulated lag phase, partial membrane rupture occurs before critical intracellular membrane modification events–perhaps those that lead to cytoskeleton collapse [26] or that provide the spontaneous membrane curvature to the erythrocyte membrane thought to be required for the elastic phenomenon described above [30, 38, 39]—have had time to go to completion. Crucially, as soon as the membranes break in the mutants, further membrane modification probably ceases due to the premature leakage and dilution of the effector molecules that mediate these modifications. These would normally function in the confined space of the PV lumen and/or host cell cytosol in the critical few minutes between SUB1 discharge and final egress. These effector molecules may include proteases such as SUB1 itself (which is released into the surrounding medium upon premature rupture of the ΔSERA5 parasites; Fig 4C), as well as SERA6 [10] and possibly host cell-derived calpain-I [40], plus putative pore-forming proteins such as PLP1 [33], which has been proposed as a candidate for mediating the cysteine protease-independent erythrocyte membrane poration that immediately precedes normal egress [32, 33], although recent gene disruption data refute this [41]. The ruptured but incompletely fragmented membranes transiently entrap the merozoites, severely impeding their efficient dissemination (Fig 8). Given the very short invasive half-life of free *P*. *falciparum* merozoites, which lies somewhere between 90–300 seconds at 37°C [29, 42–45], it is likely that any delay in the dispersal of free merozoites from the bounding membrane(s) of a ruptured schizont impacts on invasion efficiency. Whilst vigorous shaking during egress increased the efficiency of invasion and ring formation, presumably due to more efficient dispersal of the released merozoites, even this could not completely rescue the phenotype. We propose that the egress defect is the primary cause of the poor replication rates observed in the ΔSERA5 and ΔSERA4/5 mutants. However, we cannot rule out other possibilities, including that the ΔSERA5 merozoites may have an intrinsic invasion defect due for example to the absence of surface-bound N- and C-terminal SERA5 processing products, which have been shown to selectively bind merozoites following SUB1-mediated cleavage [9]. Unfortunately, distinguishing between these alternative models is experimentally challenging, and attempts by us to assess the invasive capacity of isolated merozoites from the ruptured ΔSERA5 schizonts were inconclusive.

We can only speculate on the molecular mechanisms underlying SERA5 function. As one of the most abundant PV proteins, SERA5 may simply act as an abundant SUB1 substrate or ‘sink’, competing with SUB1-mediated cleavage of other key substrates in the moments following SUB1 discharge and thus slowing down the kinetics of the egress pathway. Arguing strongly against this is our inability to discern any changes in the rate of processing of the known SUB1 substrates MSP1 and SERA6 in the ΔSERA5 mutants (S3 Fig). An alternative model rests on the fact that SERA5 bears structural resemblance to a cysteine protease [2] and indeed displays peptidase activity when the ‘active site’ Ser596 is replaced by a Cys residue [6]. Catalytically inactive enzyme orthologues, including pseudokinases and pseodoproteases, are common, often thought to have evolved from their catalytically active cognate enzymes [46–49] and sometimes existing in regulatory complexes with them [50, 51]. SERA5 may likewise act in concert with a related partner enzyme. A good candidate partner is the egress-related enzyme SERA6, a proposition supported by phylogenetic analyses suggesting that the ancestral gene from which all the Ser-type *SERA* genes evolved was a Cys-type gene likely corresponding to either SERA6 or SERA7 in *P*. *falciparum* [3]. However, exhaustive attempts by us to identify interactions between SERA5 and SERA6 or any other candidate partners by co-immunoprecipitation have been unsuccessful; for example, we can readily obtain chromatographically pure SERA5 from parasite extracts in both full-length [17] and processed form [6] under non-denaturing conditions at neutral pH, suggesting that SERA5 does not form high affinity associations with other soluble parasite or host proteins. A third model for SERA5 function, related to the second, is that SERA5 might act by binding and protecting substrates of one or more egress-related proteases, transiently inhibiting cleavage of those substrates. The binding of SERA5 might be released by SUB1 and/or protease X-mediated cleavage of SERA5, explaining the importance of processing for SERA5 function. Notably, either of these pseudoprotease roles for SERA5 would be consistent with our observation in this study that in the presence of E64 the rate of progression to PVM rupture was independent of SERA5 expression. This is because if the function of SERA5 is to control the enzymatic rate of a related cysteine protease against critical physiological substrates, then pharmacological inhibition of that cysteine protease would be expected to negate the effects of loss of SERA5. Elucidating the molecular details of SERA5-mediated regulation will be a key priority of further work.

Prior to this work, all attempts to directly disrupt the *P*. *falciparum SERA5* gene using conventional targeted homologous recombination had failed [4–6], leading to the conclusion that the gene is indispensable for *in vitro* growth. Our results here, using a robust conditional gene modification system, clearly demonstrate that this is not the case, although we also show that due to their replication defect ΔSERA5 parasites can be rapidly out-competed *in vitro* by wild type parasites. We suspect that the previous lack of success in obtaining SERA5-null parasites was due to the inefficient nature of homologous recombination in *P*. *falciparum*, combined with the technical difficulties of isolating blood stage mutants with a growth defect because of their selective disadvantage in the presence of wild-type parasites. We expect that future work using DiCre and similar efficient conditional approaches will enable the isolation of many malarial mutants lacking expression of genes previously considered to be essential. As in this case, the resulting phenotypes will provide valuable insights into the biology of this important pathogen.

## Materials and methods

### Reagents and antibodies

The antifolate drug WR99210 was from Jacobus Pharmaceuticals (New Jersey, USA). Blasticidin, E64 and rapamycin were from Sigma. Rapamycin was used as described previously [20]. The PKG inhibitor compound 1 was a kind gift of David Baker, London School of Hygiene & Tropical Medicine, whilst compound 2 was kindly provided by Dr Simon Osborne, MRC Technology, London NW7 1AA, UK. Stocks of both drugs were stored in dry DMSO at -20°C, and were used throughout at final concentrations of 2 μM (compound 1) and 1 μM (compound 2). Monoclonal antibody (mAb) 89.1, which recognises the merozoite surface protein MSP1, has been described previously [52], as has the human anti-MSP1 mAb X509 [53], rabbit polyclonal antisera to *P*. *falciparum* SERA5 [6] and SERA6 [10], and a rabbit polyclonal antiserum against *P*. *falciparum* SUB1 [54]. The anti-SERA5 mAb NIMP.M13 was produced using a previously described method [18], using B-cells from BALB/c mice (bred in the pathogen-free animal facility of the Medical Research Council National Institute for Medical Research, Mill Hill, London) immunised with recombinant full-length SERA5 [6]. Rabbit antibodies specific to relatively non-conserved regions of *P*. *falciparum* SERA3 and SERA4 (anti-SE3N and anti-SE4N) were kind gifts of Professor Toshihiro Horii, Osaka University, Japan.

### *P*. *falciparum* culture, transfection and limiting dilution cloning

Asexual blood stages of the DiCre-expressing *P*. *falciparum* clone 1G5DC [20] were cultured in an atmosphere of 90% nitrogen, 5% carbon dioxide and 5% oxygen at 37°C in RPMI 1640 medium containing Albumax (Invitrogen) supplemented with 2 mM L-glutamine, and synchronised using standard procedures [55, 56]. Parasite developmental stage and viability was routinely assessed by microscopic examination of Giemsa-stained thin blood films. For transfection of integration constructs, Percoll-enriched synchronised mature schizonts were electroporated with plasmid DNA (10 μg per transfection) using an Amaxa P3 primary cell 4D Nucleofector X Kit L (Lonza) as described [27]. Growth medium was replaced ~20 h post transfection with fresh medium containing 2.5 nM WR99210. Once drug-resistant parasites appeared and displayed robust growth (2–3 weeks post-transfection), they were subjected to repeated cycles of culture for 3 weeks without drug followed by culturing with drug (drug cycling) to select for parasites in which integration into the genome had taken place [56]. Transgenic parasite clones floxSERA5-1B6, floxSERA5-3B6 and floxSERA4/5-B52 were obtained by limiting dilution cloning in round-bottomed wells at a calculated 0.1–0.3 parasite per well as described [56]. Following RAP-treatment of these parasites to induce gene disruption, clones of ΔSERA5 or ΔSERA4/5 parasites were obtained by limiting dilution in flat-bottomed 96-well microplate wells as described [7], plating a calculated 10 parasites per well. Only wells containing single plaques were subsequently used. Once established, all transgenic clones except the parental 1G5DC clone were maintained in medium containing 2.5 nM WR99210.

### Immunofluorescence and western blot

For IFA, air-dried thin films of parasite cultures were fixed in paraformaldehyde, permeabilized, then probed with relevant primary antibodies as described previously [10]. Secondary Alexa Fluor 488 or 594-conjugated antibodies specific for human, rabbit or mouse IgG (Invitrogen) were used at a dilution of 1:10,000. Samples were stained with 4,6-diamidino-2-phenylindol (DAPI) for nuclear staining then mounted in Citifluor (Citifluor Ltd., UK). Images were acquired using a Zeiss Axioplan 2 Imaging system (Carl Zeiss, Germany) and AxioVision 3.1 software, using identical exposure conditions for all samples being compared. Western blots were prepared and probed as described previously [57].

### Production of transgenic *P*. *falciparum* for conditional disruption of the *SERA5* or *SERA4* plus *SERA5* genes

A second *loxP* site was introduced into the 1G5DC genome upstream of the *SERA5* locus by targeting it to a site just downstream of the *SERA4* gene. To do this, plasmid pHH1_S4int_US-loxP was first generated by excising the fragment containing the *loxP* site immediately upstream of the *SERA5* targeting region in plasmid pHH1_PreDiCre_A [20] using BamHI and HpaI. This fragment was ligated into pHH1-ΔSERA4 [4] pre-digested with the same restriction enzymes. To produce the targeting sequences, PCR products were amplified from 3D7 genomic DNA using Phusion HF DNA polymerase (NEB) with forward primers S4_F3 or S4_HpaI_F and reverse primer S4_XhoI_R, generating target regions 1 (~1.4 kb) and 2 (~1 kb), respectively. The PCR products were blunt-ended using T4 DNA polymerase (NEB) then digested with XhoI and cloned into plasmid pHH1_S4int_US-loxP pre-digested with HpaI and XhoI to remove the internal *SERA4* sequence, giving rise to plasmids pSERA4loxPa and pSERA4loxPb, respectively. The plasmids were independently transfected into mature 1G5DC schizonts and WR99210-resistant parasites subjected to drug cycling to select for integration into the genome. The resulting parasite lines were screened by diagnostic PCR using primers S4_F4 plus S4_DS_R1 to detect the unmodified 1G5DC *SERA5* locus (giving rise to a 1985 bp fragment) and primers CAM5’_R3 plus S4_DS_R1 to detect the integrated locus (giving rise to a 1941 bp fragment for target fragment 1, or a 1535 bp fragment for target fragment 2). Limiting dilution cloning of the integrated parasite lines resulted in clones floxSERA5-1B6 (from integration using targeting fragment 2) and floxSERA5-3B6 (from integration using targeting fragment 1).

A transgenic *P*. *falciparum* line for simultaneous conditional disruption of the *SERA4* and *SERA5* genes was generated by introducing a *loxP* site downstream of the *SERA3* locus in the 1G5DC parasite clone, thus flanking both *SERA4* and *SERA5* genes simultaneously. To do this the *SERA3* targeting region was amplified using forward primer S3_F1 and reverse primer S3_R1 to generate a 1.4 kb product. This was digested with SnaBI and XhoI and cloned into pHH1_S4int_US-loxP, pre-digested with HpaI and XhoI, generating construct pSERA3loxP. This was transfected into 1G5DC schizonts and drug-selected parasites screened for integration using primers CAM5’_R3 and S3_DS_R1 (which produce an amplicon of ~1.7 kb). The presence of the endogenous locus was detected using primers S3_F6 and S3_DS_R1 giving rise to a product of 2.0 kb. Limiting dilution cloning of the integrant parasite population generated parasite clone floxSERA4/5-B52.

For both the *SERA5* and *SERA4/5* gene disruption experiments, following treatment of parasites with RAP (100 nM for 1–4 h at 37°C) the non-excised locus was detected by diagnostic PCR using GoTaq Green (Promega), using primers sgS5_seq4F and hsp86_3’_R1 (amplicon size ~1200 bp) whilst the excised locus was detected using primers CAM5’_R3 and hsp86_3’_R1 (amplicon size 580 bp). The ΔSERA5 *P*. *falciparum* clones 2F8_ΔSERA5 and C6_ΔSERA5 were selected from RAP-treated conditional knockout floxSERA5-1B6 and floxSERA5-3B6 respectively. The ΔSERA4/5 clones C10_ΔSERA4/5 and G8_ΔSERA4/5 were similarly obtained by limiting dilution cloning of RAP-treated clone floxSERA4/5-B52.

### Parasite growth and plaque assays

Parasitaemia measurements by FACS were as described previously [6]. Briefly, parasites recovered at various time-points were stained with the fluorescent vital stain hydroethidine. As negative controls, uninfected erythrocytes in culture medium were stained and processed in the same way. Parasitaemia was calculated using the FACSCalibur flow cytometer (Becton Dickson) as described previously [58]. Briefly, cultures to be analysed were initially screened using forward and side scatter parameters and gated for erythrocytes. From this gated population, the proportion of HE-stained cells in 100,000 cells was determined using the FL2 detector (585/42 nm).

Plaque assays were performed by dispensing parasite cultures in flat-bottomed microplates at a haematocrit of 0.75%, as described [7]. Plates were imaged using a high resolution flat-bed scanner 14–16 days after setting up the assays. Plaques were counted by visual examination of the images and plaque size quantified using the Lasso tool in Adobe Photoshop CS6. Statistical analysis (linear regression analysis by analysis of covariance and *t*-test) was performed using GraphPad Prism 7 software (CA, USA) or tools available on the GraphPad calculation website (http://www.graphpad.com/quickcalcs/ttest1/). When required, parasites from wells containing a single plaque were expanded by transferring initially to round-bottomed microplate wells, before further expansion into culture flasks.

### Complementation plasmid construction and transfection

For transgenic expression of SERA5 or mutants thereof in SERA5-null parasites, a SERA5 expression cassette under the regulation of its native promoter was initially assembled in pHH1_preDiCre_A [20]. This plasmid already contained the 3’ fragment of a previously described synthetic recodonised *SERA5* gene, called *SERA5*_s*ynth*_ [20]. The 5’ region of the *SERA5* synthetic gene was excised from N-term-pMK by digesting with Pac1 and blunt ending with T4 DNA polymerase, followed by digestion with SalI. The resulting fragment was cloned into pHH1_preDiCre_A pre-digested with HpaI and SalI giving rise to plasmid pHH1_*sg-sera5*. The *SERA5* promoter region was then amplified from 3D7 genomic DNA with primers S5_US_F5 and S5_US_R3 using Phusion HF DNA polymerase and cloned into pHH1_*sg-sera5* using AflII and SnaBI sites, giving rise to plasmid pHH1_S5-5’_sgS5. To distinguish the synthetic gene product from that of the endogenous gene, a mini TAP tag was incorporated into the *SERA5* coding region just downstream of the P50C (protease X) cleavage site [6] by cloning a SbfI and XmaI fragment including the mini TAP tag from pHH1SERA5chimΔP6TAP [6] into pHH1_S5-5’_sgS5, generating plasmid pHH1_S5-5’_sgS5_mT. To obtain constitutive expression of mCherry from the *SERA5* expression plasmid, the *SERA5* expression cassette was then excised from pHH1_S5-5’_sgS5_mT using NotI-HF and SnaBI, blunt-ended with T4 DNA polymerase, and cloned into pDC2-mCherry (a kind gift of Catherine Suarez, The Francis Crick Institute) pre-digested with BamHI and blunt-ended using T4 DNA polymerase, giving rise to the wild-type complementation plasmid pDC2_mC_sgS5.

Mutagenesis of the SUB1 site 1 cleavage of SERA5 was introduced by carrying out two separate PCR reactions using primers sgS5_5’_F_AflII plus S5_st1II_R, and S5_st1II__F plus PbDT3’_R1. This was followed by an overlapping PCR reaction in which the two PCR products were mixed and amplification carried out using the external primers sgS5_5’_F_AflII and PbDT3’_R1. The resulting mutated DNA fragment was cloned into pHH1_S5-5’_sgS5_mT using BglII and AflII sites, giving rise to plasmid pHH1_S5-5’_sgS5/st1_mT.

The SUB1 site 2 cleavage site of SERA5 was removed when the mini TAP tag was introduced into the *SERA5* sequence [6]. To additionally mutate the protease X (P50C) cleavage site, two separate PCR reactions were carried out using primers S5synF1 plus p50_R_New, and p50_F_New plus PbDT3’_R1. This was followed by an overlapping PCR using primers S5synF1 and PbDT3’_R1. The resulting mutant amplicon was sub-cloned into pHH1_S5-5’_sgS5_mT using BglII and AvrII giving rise to plasmid pHH1_S5-5’_sgS5/p50+st2_mT. To produce a modified SERA5 gene containing mutations at both the SUB1 sites 1 and 2 as well as the P50C cleavage site, the SUB1 site 1 cleavage site mutation from pHH1_S5-5’_sgS5/st1_mT was cloned into plasmid pHH1_S5-5’_sgS5/p50+st2_mT using XcmI. The orientation of the insert was confirmed by sequencing, then the mutant DNA fragment sub-cloned into plasmid pDC2_mC_sgS5 using SnaBI and NotI giving rise to the final mutant complementation plasmid pDC2_mC_sgS5mut.

For introduction of the complementation and control constructs into parasites, mature schizonts of the ΔSERA5 *P*. *falciparum* clone 2F8_ΔSERA5 were electroporated as described above with 10 μg of each construct pDC2_mC_sgS5, pDC2_mC_sgS5mut or pDC2_mCherry and maintained initially in medium containing no drug. Approximately 24 h following electroporation, the parasites were transferred into medium supplemented with blasticidin (2 μg ml^-1^) then maintained until vigorous growth ensued. All lines were synchronised before use for Western blot, video microscopy or growth assays.

### Time-lapse DIC and fluorescence video microscopy

Viewing chambers (internal volume ~80 μl) for observation of live schizonts were constructed as described [18] by adhering 22 x 64 mm borosilicate glass coverslips to microscope slides with strips of double-sided tape, leaving ~4 mm gaps at each end. Schizont samples were washed then suspended in warm, gassed complete medium, either alone or supplemented where required with E64 (50 μM final concentration), Alexa Fluor 488 phalloidin (Invitrogen; diluted 1:50 from a 200 unit ml^-1^ stock in methanol), and/or Alexa Fluor 647-conjugated wheat germ agglutinin (Thermo Fisher; diluted 1:1000 from a 1 mg ml^-1^ stock in phosphate-buffered saline).The schizont suspension was introduced into the pre-warmed chamber, the ends were sealed and the slide transferred to a temperature-controlled microscope stage held at 37°C. Images were taken either on a Zeiss Axio Imager M1 microscope equipped with an EC Plan-Neofluar 100x/1.3 oil immersion DIC objective and an AxioCam MRm camera, or on a Nikon Eclipse Ni-E widefield microscope fitted with a Hamamatsu C11440 digital camera and Nikon N Plan Apo λ 100x/1.45NA oil immersion objective. Images were taken at 5 s intervals over a total of 20–30 min, then annotated and exported as TIFFs, AVI or QuickTime movies using Axiovision 3.1 or Nikon NIS-Elements software. Mean fluorescence intensity values of individual mCherry-expressing schizonts were determined from exported raw image files (TIFF format) as described previously [18], using the Lasso tool and Histogram options of Adobe Photoshop CS6.

## Supporting information

S1 Fig Efficient and selective DiCre-mediated ablation of SERA5 expression in the floxSERA5-1B6 and floxSERA5-3B6 *P. falciparum* clones. (A) IFA of mature schizonts of the two integrant parasite clones ~44 h following RAP-treatment. Samples were probed with the anti-SERA5 mAb NIMP.M13 and the anti-MSP1 human mAb X509. Microscopic counts showed that only 1.67±0.13% of the RAP-treated floxSERA5-1B6 parasites and 2.1±0.61% of the RAP-treated floxSERA5-3B6 parasites showed detectable SERA5 expression (n≥1500 in each case). The fields of view shown here were deliberately selected in order to show examples of the rare residual SERA5-expressing parasites alongside ΔSERA5 parasites. Scale bar, 5 μm. (B) Western blot quantitation of SERA5 expression levels in mock-treated and RAP-treated floxSERA5-1B6 populations shows an overall reduction in SERA5 expression of >95%. The same mAbs were used to probe as in (A), with the antibody to MSP1 acting as a loading control. Equal volumes (10 μl) of serially-diluted SDS extract from identical numbers of parasites were loaded per track. (C) Western blot showing that DiCre-mediated ablation of SERA5 expression had no discernible effects on expression of SERA4 and SERA6. SERA-specific rabbit antibodies were used to probe the blots. (D) Giemsa-stained images of RAP-treated (ΔSERA5) and mock-treated floxSERA5-1B6 schizonts ~44 h following treatment. Scale bar, 5 μm. (E) Coomassie-stained SDS PAGE gel of SDS extracts of mock-treated or RAP-treated floxSERA5-1B6 schizonts. The only detectable difference was the absence of a ~120 kDa species from the RAP-treated extract, identified by Western blot as SERA5. Positions of pre-stained molecular mass marker proteins (left-hand track) are indicated.
(JPG)

S2 Fig Loss of SERA5 expression results in accelerated but defective egress. (A) Stills from time-lapse DIC microscopic imaging showing stages in rupture of mock (DMSO)-treated (control) and RAP-treated (ΔSERA5) schizonts of *P*. *falciparum* clone floxSERA5-1B6. Movies were started exactly 3 min following removal of the reversible PKG inhibitor compound 1 (time following washing away the inhibitor is indicated). The ΔSERA5 parasites underwent accelerated membrane rupture (examples are labelled with white arrows) with only gradual dispersal of the merozoites, unlike the ‘explosive’ egress typical of control parasites. The total proportion of observed schizonts that underwent rupture in the two populations over the imaging period was 35% (28 of 80) for the mock-treated parasites and 34% (51 of 148) for the RAP-treated population. (B) Quantitation of the timing of membrane rupture in the control and ΔSERA5 parasites. Whereas egress in the control parasites did not take place before 13 min, most of the membrane rupture evident in the ΔSERA5 parasites had already occurred by that point. Time to egress is indicated to the nearest 0.5 min. Data were collated from visual examination of frames from 2–3 videos each of mock and RAP-treated clone floxSERA5-1B6 (total number of egress events: RAP-treated, 51; mock-treated, 28). Time to egress statistics were calculated for the RAP-treated parasites (mean 10.5 min, SD 5.6 min) and for the control parasites (mean 18.8 min, SD 3.2 min), with a two-tailed unpaired *t*-test revealing the difference to be extremely significant (t = 7.2297, d.f. = 77, p <0.0001). (C) The residual host red blood cell membrane remains associated with ΔSERA5 merozoite clusters. Two sets of example stills from simultaneous time-lapse DIC and fluorescence microscopic imaging showing ΔSERA5 schizonts surface-labelled with Alexa Fluor 647-conjugated wheat germ agglutinin (WGA; cyan) prior to and following rupture. The presence of fragmented residual host erythrocyte membranes closely associated with the resulting merozoite clusters is clearly visible. Scale bar, 10 μm.
(TIF)

S3 Fig Loss of SERA5 expression has no effect on the rate of proteolytic processing of SUB1 substrates MSP1 and SERA6. Western blot analysis of control and RAP-treated (ΔSERA5) schizonts of *P*. *falciparum* clone floxSERA5-1B6, sampled within minutes (indicated) of release from a compound 2-mediated egress block. Blots were probed with antibodies to (A) MSP1 or (B) SERA6, both established SUB1 substrates. SUB1-mediated processing of SERA6 results in its apparent disappearance because the antiserum used does not recognise the processed products [10]. In contrast, the anti-MSP1 antibody used here (mAb 89.1) recognizes both the full-length MSP1 and a doublet corresponding to the processed product MSP1_83_ (indicated). Loss of SERA5 expression had no detectable effect on the rate of proteolytic processing of either protein.
(JPG)

S4 Fig Loss of SERA5 expression has no effect on the timing of PVM rupture in the presence of the cysteine protease inhibitor E64. (A) Stills from time-lapse DIC microscopic imaging showing time points leading up to and following PVM rupture in a RAP-treated (ΔSERA5) schizont of *P*. *falciparum* clone floxSERA5-3B6 in the presence of E64 (50 μM). The point of PVM rupture (arbitrarily set to zero seconds) is clearly distinguishable by the sudden loss of differential interference contrast and increased merozoite visibility. Scale bar, 10 μm. (B) Quantitation of the timing of PVM rupture in the control and ΔSERA5 schizonts in the presence of E64 following removal of the reversible PKG inhibitor compound 2. Data were collated from visual examination of frames from time-lapse DIC videos of mock and RAP-treated clone floxSERA5-3B6. Times are indicated to the nearest 0.5 min and all movies were started exactly 4.5 min following washing away the inhibitor. Time to PVM rupture statistics were calculated for the ΔSERA5 parasites (mean 17.0 min, SD 7.3 min) and for the control parasites (mean 16.9 min, SD 6.8 min), with a two-tailed unpaired *t*-test revealing the difference to be not significant (t = 0.0584, d.f. = 121, p = 0.9536).
(JPG)

S5 Fig DiCre-mediated conditional disruption of both the *P. falciparum SERA4* and *SERA5* genes. (A) Strategy for simultaneous conditional deletion of both the *SERA4* and *SERA5* genes. Targeting construct pSERA3loxP contains ~1.4 kb of 3´ *SERA3* sequence to drive integration of the entire construct into the *P*. *falciparum* clone 1G5DC *SERA3* locus by single-crossover homologous recombination. For clarity, the intron-exon structure of the *SERA* genes is not indicated. The targeting sequence extended to and included the *SERA3* stop codon and was followed by the 3′ UTR of the *P*. *berghei* dihydrofolate reductase (P*bdt*) gene to ensure correct regulation of the modified *SERA3* gene. Correct integration was expected to reconstitute the gene whilst introducing a *loxP* site downstream of the introduced *hdhfr* selection cassette. DiCre-mediated recombination was predicted to excise the entire sequence between the *loxP* sites, including both the *SERA4* and *SERA5* genes. Positions of hybridisation of primers used for diagnostic PCR analysis of integration and excision events are shown as black arrows. Primer identities are: a, S3_F6; b, S3_DS_R1; c, CAM5´_R3; d, hsp86_3´_R1; e, sgS5_seq4F; f, S3_F7; g, S3_R1 (see S1 Table for sequences of all primers used in this study). (B) Diagnostic PCR analysis of genomic DNA from the parental 1G5DC *P*. *falciparum* clone and integrant clone floxSERA4/5-B52, confirming the predicted integration event. Expected sizes of the PCR amplicons are indicated. (C) PCR analysis of genomic DNA from RAP-treated or control parental integrant and two ΔSERA4/5 *P*. *falciparum* clones, confirming the predicted DiCre-mediated excision events. The expected sizes of the PCR amplicons specific for the intact, excised and control locus are indicated.
(JPG)

S6 Fig Mutations introduced into an episomally-expressed *SERA5* gene to prevent its proteolytic processing. Schematic representation of the 3D7 *P*. *falciparum* SERA5 primary structure with the major processing fragments derived from SUB1 cleavage (P47, P56 and P18) indicated. The P56 fragment is trimmed near its C-terminus (to form the P50 terminal processing product; not depicted on this schematic) by further cleavage at the P50C cleavage site, mediated by an unknown cysteine protease called protease X [6]. The wild-type sequence flanking these sites is shown, alongside the modifications made (shown in red) by introduction of a mini TAP tag (major elements of which are also underlined and indicated) as well as introduction of several substitutions at the P50C and SUB1 site 1 cleavage sites. Note that insertion of the mini TAP tag sequence modifies the SUB1 site 2 cleavage site but has previously been shown to be tolerated when introduced into the endogenous *SERA5* gene [6]. All the modifications shown in red were present in the mutant *SERA5* gene expressed in plasmid construct pDC2_mC_sgS5mut, whereas only the mini TAP tag sequence was present in the pDC2_mC_sgS5 construct.
(JPG)

S1 Movie ΔSERA5 parasites display an egress defect. Mock-treated or RAP-treated floxSERA5-1B6 parasites were allowed to mature to schizont stage then further incubated for 4 h in the presence of compound 2 (1 μM). The parasites were washed, resuspended in fresh warm medium without compound 2 and observed by time-lapse DIC microscopy, starting imaging precisely 4.5 min following the first wash. Time from start of imaging is indicated (top left-hand side) in minutes. In the video shown, as well as other videos of the same parasite populations, the total proportion of observed schizonts that ruptured over the imaging period was 67.9% (178 of 262) for the control parasites and 79.2% (179 of 226) for the ΔSERA5 population.
(WMV)

S2 Movie ΔSERA5 parasites display an egress defect. Synchronous floxSERA5-1B6 parasites were RAP-treated at ring stage, allowed to mature to schizont stage then returned to culture (without PKG inhibitors) to allow further maturation and egress. Samples were then transferred directly to a viewing chamber and observed by time-lapse DIC microscopy. Time from start of imaging is indicated (top left-hand side) in minutes. Clusters of merozoites typical of abortive rupture are obvious, as well as a single abortive egress event (centre of video at ~17.5 min).
(MOV)

S3 Movie RAP-treatment of the parental 1G5DC *P. falciparum* clone has no effect on egress. A synchronous culture of 1G5DC parasites were RAP-treated at ring stage, allowed to mature to schizont stage then further incubated for 4 h in the presence of compound 1 (2 μM). The parasites were washed, resuspended in fresh warm medium without compound 1 and observed by time-lapse DIC microscopy, starting imaging precisely 4.5 min following the first wash. Time from start of imaging is indicated (top left-hand side) in minutes.
(AVI)

S4 Movie The ΔSERA5 *P. falciparum* clone 2F8_ΔSERA5 displays an accelerated and abortive egress defect. 2F8_ΔSERA5 parasites were allowed to mature to schizont stage then further incubated for 4 h in the presence of the PKG inhibitor compound 2 (1 μM). The parasites were washed, resuspended in fresh warm medium without compound 2 and observed by time-lapse DIC microscopy, starting imaging precisely 4.5 min following the first wash. Time from start of imaging is indicated (top left-hand side) in minutes.
(AVI)

S5 Movie The ΔSERA5 *P. falciparum* clone C6_ΔSERA5 displays an accelerated and abortive egress defect. C6_ΔSERA5 parasites were allowed to mature to schizont stage then further incubated for 4 h in the presence of the PKG inhibitor compound 2 (1 μM). The parasites were washed, resuspended in fresh warm medium without compound 2 and observed by time-lapse DIC microscopy, starting imaging precisely 4.5 min following the first wash. Time from start of imaging is indicated (top left-hand side) in minutes.
(AVI)

S6 Movie ΔSERA5 parasites are not defective in erythrocyte membrane poration. Synchronous ring stages of the floxSERA5-3B6 clone were RAP-treated, allowed to mature to schizont stage then further incubated for 4 h in the presence of compound 2 (1 μM). The parasites were washed, transferred to fresh medium containing Alexa Fluor 488-labeled phalloidin plus E64 (50 μM), then imaged by simultaneous time-lapse DIC and fluorescence imaging. The appearance of phalloidin labelling in individual schizonts (at ~16.0 min in this movie) was always temporally coincident with PVM rupture, visualised as the sudden loss of differential interference contrast of the schizont and increased visibility and mobility of the intracellular merozoites.
(WMV)

S7 Movie Normal egress by control schizonts of the floxSERA4/5-B52 clone. A synchronous culture of floxSERA4/5-B52 parasites was mock-treated (with 0.1% v/v DMSO) at ring stage and allowed to mature to schizont stage. Schizonts were then further incubated for 4 h in the presence of compound 1 (2 μM). The parasites were washed, transferred to fresh medium, and observed by time-lapse DIC microscopy, starting imaging precisely 4.5 min following the first wash. Time from start of imaging is indicated (top left-hand side) in minutes. Normal explosive egress is observed in the majority of schizonts.
(WMV)

S8 Movie ΔSERA4/5 parasites display an egress defect. A synchronous culture of floxSERA4/5-B52 parasites was RAP-treated at ring stage, allowed to mature to schizont stage then further incubated for 4 h in the presence of compound 1 (2 μM). The parasites were washed, transferred to fresh medium, and observed by time-lapse DIC microscopy, starting imaging precisely 4.5 min following the first wash. Time from start of imaging is indicated (top left-hand side) in minutes. Accelerated, abortive rupture is observed starting from ~2 min following beginning of imaging.
(MOV)

S9 Movie Episomal expression of an epitope-tagged wild-type *SERA5* gene rescues the ΔSERA5 abortive egress phenotype. Simultaneous live DIC and fluorescence time-lapse imaging of mature schizonts of the 2F8_ΔSERA5:SERA5wt parasite line following release of a compound 2-mediated block. The mCherry-positive schizonts (indicating the presence of the complementing episome) display normal egress, whilst abortive egress typical of ΔSERA5 parasites is seen in the other schizonts.
(MP4)

S10 Movie Episomal expression of an epitope-tagged mutant (cleavage-defective) *SERA5* gene fails to rescue the ΔSERA5 abortive egress phenotype. Simultaneous live DIC and fluorescence time-lapse imaging of mature schizonts of the 2F8_ΔSERA5:SERA5mut parasite line following release of a compound 2-mediated block. The mCherry-positive schizonts (indicating the presence of the complementing episome) display abortive egress typical of ΔSERA5 parasites.
(MP4)

S1 Table Oligonucleotide primer sequences used in this study. (PDF)
