# Supplementary figures and images for: The Plasmodium falciparum pseudoprotease SERA5 regulates the kinetics and efficiency of malaria parasite egress from host erythrocytes
Source: PLoS Pathog. 2017 Jul 6;13(7):e1006453. doi: 10.1371/journal.ppat.1006453 (PMC5500368; doi:10.1371/journal.ppat.1006453)

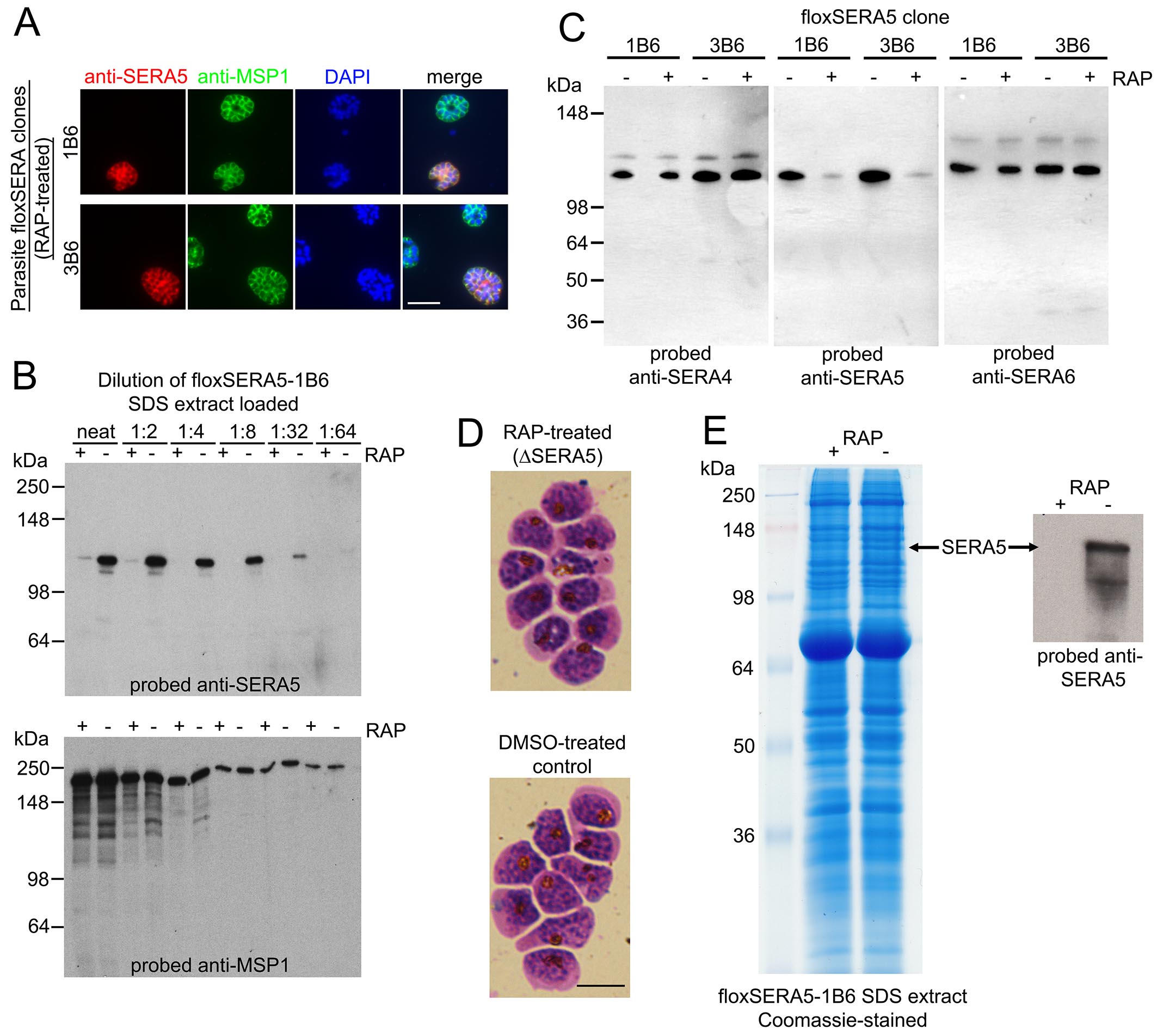

Supplement: S1 Fig — (A) IFA of mature schizonts of the two integrant parasite clones ~44 h following RAP-treatment. Samples were probed with the anti-SERA5 mAb NIMP.M13 and the anti-MSP1 human mAb X509. Microscopic counts showed that only 1.67±0.13% of the RAP-treated floxSERA5-1B6 parasites and 2.1±0.61% of the RAP-treated floxSERA5-3B6 parasites showed detectable SERA5 expression (n≥1500 in each case). The fields of view shown here were deliberately selected in order to show examples of the rare residual SERA5-expressing parasites alongside ΔSERA5 parasites. Scale bar, 5 μm. (B) Western blot quantitation of SERA5 expression levels in mock-treated and RAP-treated floxSERA5-1B6 populations shows an overall reduction in SERA5 expression of >95%. The same mAbs were used to probe as in (A), with the antibody to MSP1 acting as a loading control. Equal volumes (10 μl) of serially-diluted SDS extract from identical numbers of parasites were loaded per track. (C) Western blot showing that DiCre-mediated ablation of SERA5 expression had no discernible effects on expression of SERA4 and SERA6. SERA-specific rabbit antibodies were used to probe the blots. (D) Giemsa-stained images of RAP-treated (ΔSERA5) and mock-treated floxSERA5-1B6 schizonts ~44 h following treatment. Scale bar, 5 μm. (E) Coomassie-stained SDS PAGE gel of SDS extracts of mock-treated or RAP-treated floxSERA5-1B6 schizonts. The only detectable difference was the absence of a ~120 kDa species from the RAP-treated extract, identified by Western blot as SERA5. Positions of pre-stained molecular mass marker proteins (left-hand track) are indicated. (JPG) [file ppat.1006453.s001.jpg]

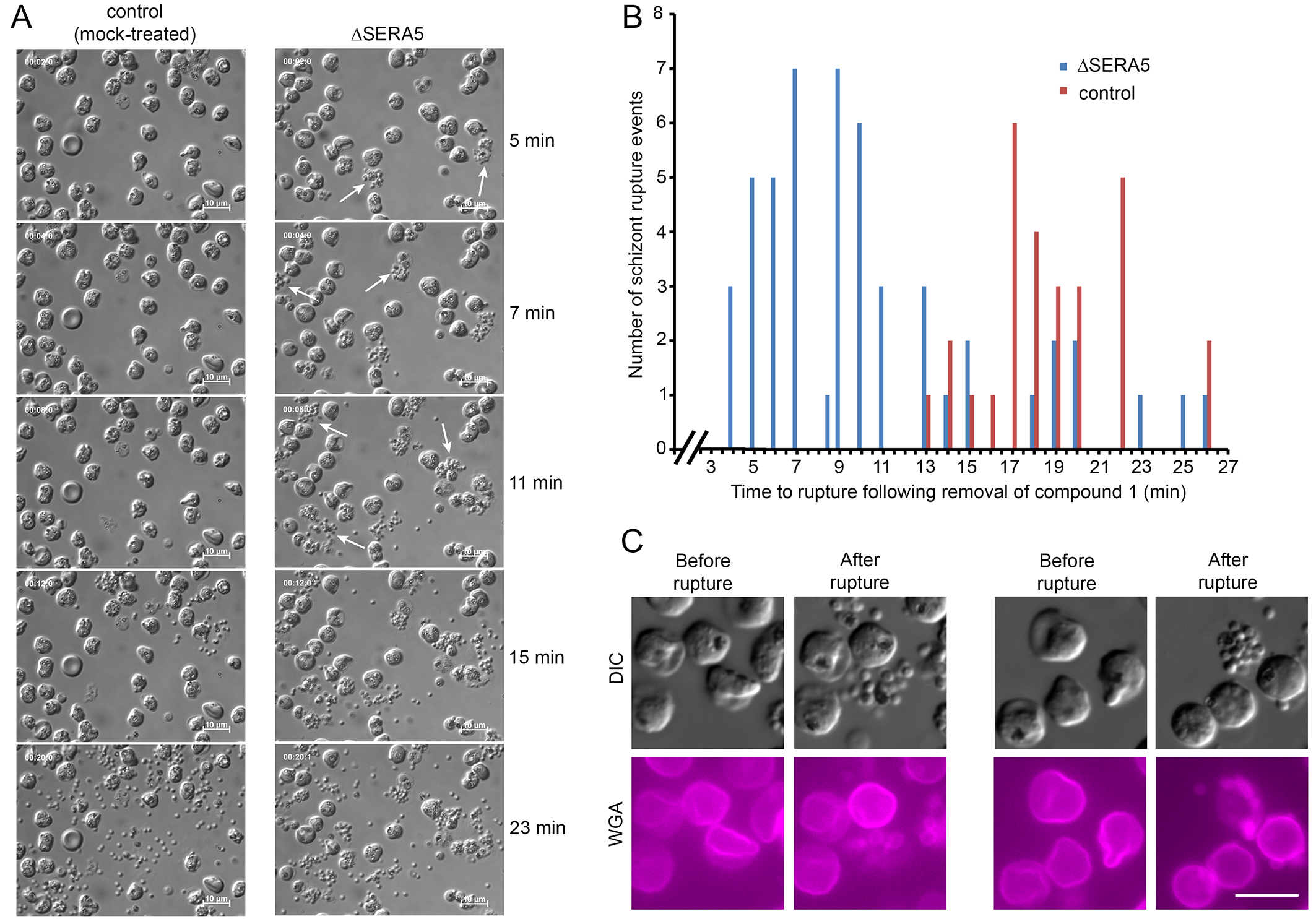

Supplement: S2 Fig — (A) Stills from time-lapse DIC microscopic imaging showing stages in rupture of mock (DMSO)-treated (control) and RAP-treated (ΔSERA5) schizonts of P. falciparum clone floxSERA5-1B6. Movies were started exactly 3 min following removal of the reversible PKG inhibitor compound 1 (time following washing away the inhibitor is indicated). The ΔSERA5 parasites underwent accelerated membrane rupture (examples are labelled with white arrows) with only gradual dispersal of the merozoites, unlike the ‘explosive’ egress typical of control parasites. The total proportion of observed schizonts that underwent rupture in the two populations over the imaging period was 35% (28 of 80) for the mock-treated parasites and 34% (51 of 148) for the RAP-treated population. (B) Quantitation of the timing of membrane rupture in the control and ΔSERA5 parasites. Whereas egress in the control parasites did not take place before 13 min, most of the membrane rupture evident in the ΔSERA5 parasites had already occurred by that point. Time to egress is indicated to the nearest 0.5 min. Data were collated from visual examination of frames from 2–3 videos each of mock and RAP-treated clone floxSERA5-1B6 (total number of egress events: RAP-treated, 51; mock-treated, 28). Time to egress statistics were calculated for the RAP-treated parasites (mean 10.5 min, SD 5.6 min) and for the control parasites (mean 18.8 min, SD 3.2 min), with a two-tailed unpaired t-test revealing the difference to be extremely significant (t = 7.2297, d.f. = 77, p <0.0001). (C) The residual host red blood cell membrane remains associated with ΔSERA5 merozoite clusters. Two sets of example stills from simultaneous time-lapse DIC and fluorescence microscopic imaging showing ΔSERA5 schizonts surface-labelled with Alexa Fluor 647-conjugated wheat germ agglutinin (WGA; cyan) prior to and following rupture. The presence of fragmented residual host erythrocyte membranes closely associated with the resulting merozoite clusters is clea [file ppat.1006453.s002.tif]

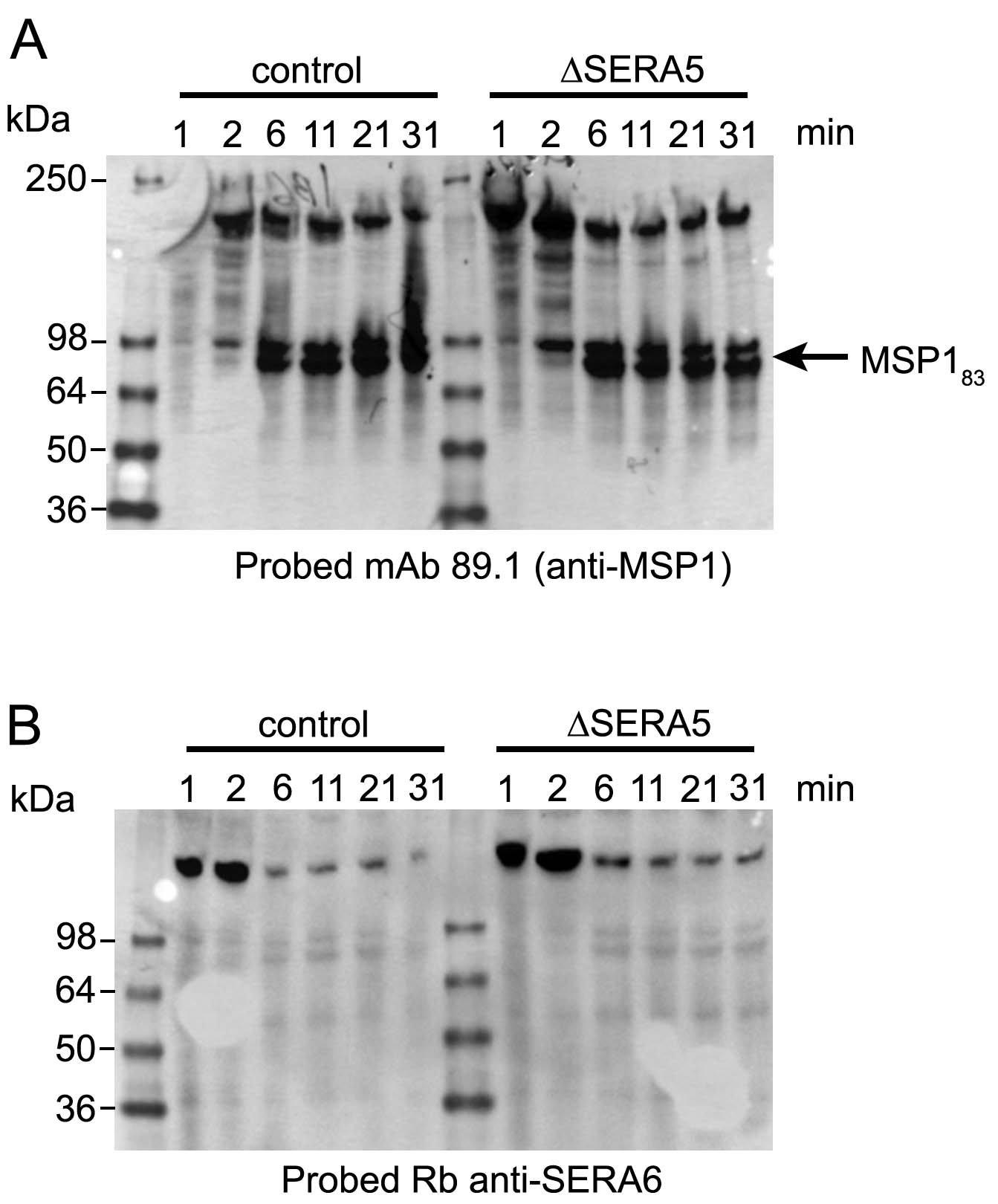

Supplement: S3 Fig — Western blot analysis of control and RAP-treated (ΔSERA5) schizonts of P. falciparum clone floxSERA5-1B6, sampled within minutes (indicated) of release from a compound 2-mediated egress block. Blots were probed with antibodies to (A) MSP1 or (B) SERA6, both established SUB1 substrates. SUB1-mediated processing of SERA6 results in its apparent disappearance because the antiserum used does not recognise the processed products [10]. In contrast, the anti-MSP1 antibody used here (mAb 89.1) recognizes both the full-length MSP1 and a doublet corresponding to the processed product MSP183 (indicated). Loss of SERA5 expression had no detectable effect on the rate of proteolytic processing of either protein. (JPG) [file ppat.1006453.s003.jpg]

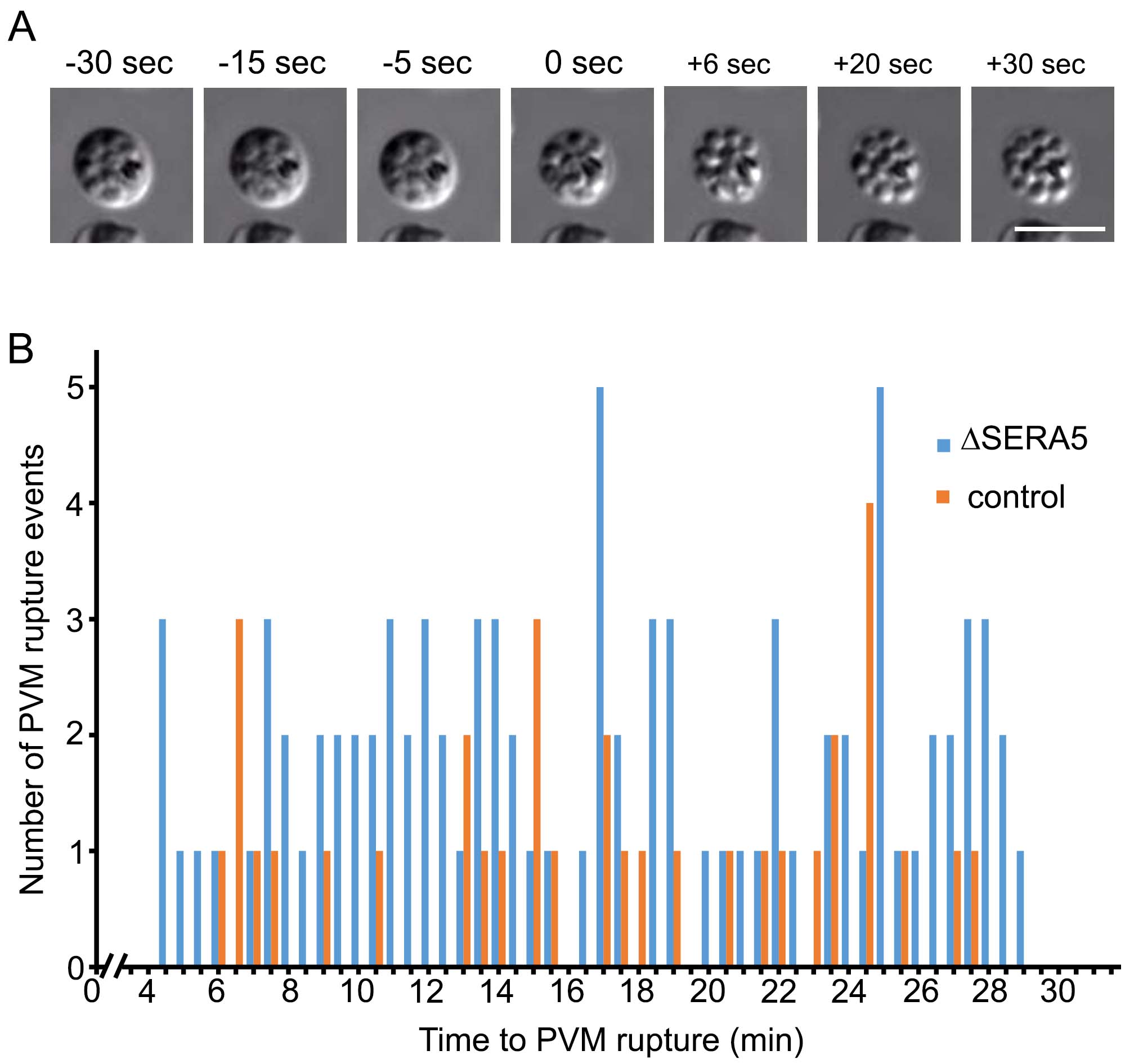

Supplement: S4 Fig — (A) Stills from time-lapse DIC microscopic imaging showing time points leading up to and following PVM rupture in a RAP-treated (ΔSERA5) schizont of P. falciparum clone floxSERA5-3B6 in the presence of E64 (50 μM). The point of PVM rupture (arbitrarily set to zero seconds) is clearly distinguishable by the sudden loss of differential interference contrast and increased merozoite visibility. Scale bar, 10 μm. (B) Quantitation of the timing of PVM rupture in the control and ΔSERA5 schizonts in the presence of E64 following removal of the reversible PKG inhibitor compound 2. Data were collated from visual examination of frames from time-lapse DIC videos of mock and RAP-treated clone floxSERA5-3B6. Times are indicated to the nearest 0.5 min and all movies were started exactly 4.5 min following washing away the inhibitor. Time to PVM rupture statistics were calculated for the ΔSERA5 parasites (mean 17.0 min, SD 7.3 min) and for the control parasites (mean 16.9 min, SD 6.8 min), with a two-tailed unpaired t-test revealing the difference to be not significant (t = 0.0584, d.f. = 121, p = 0.9536). (JPG) [file ppat.1006453.s004.jpg]

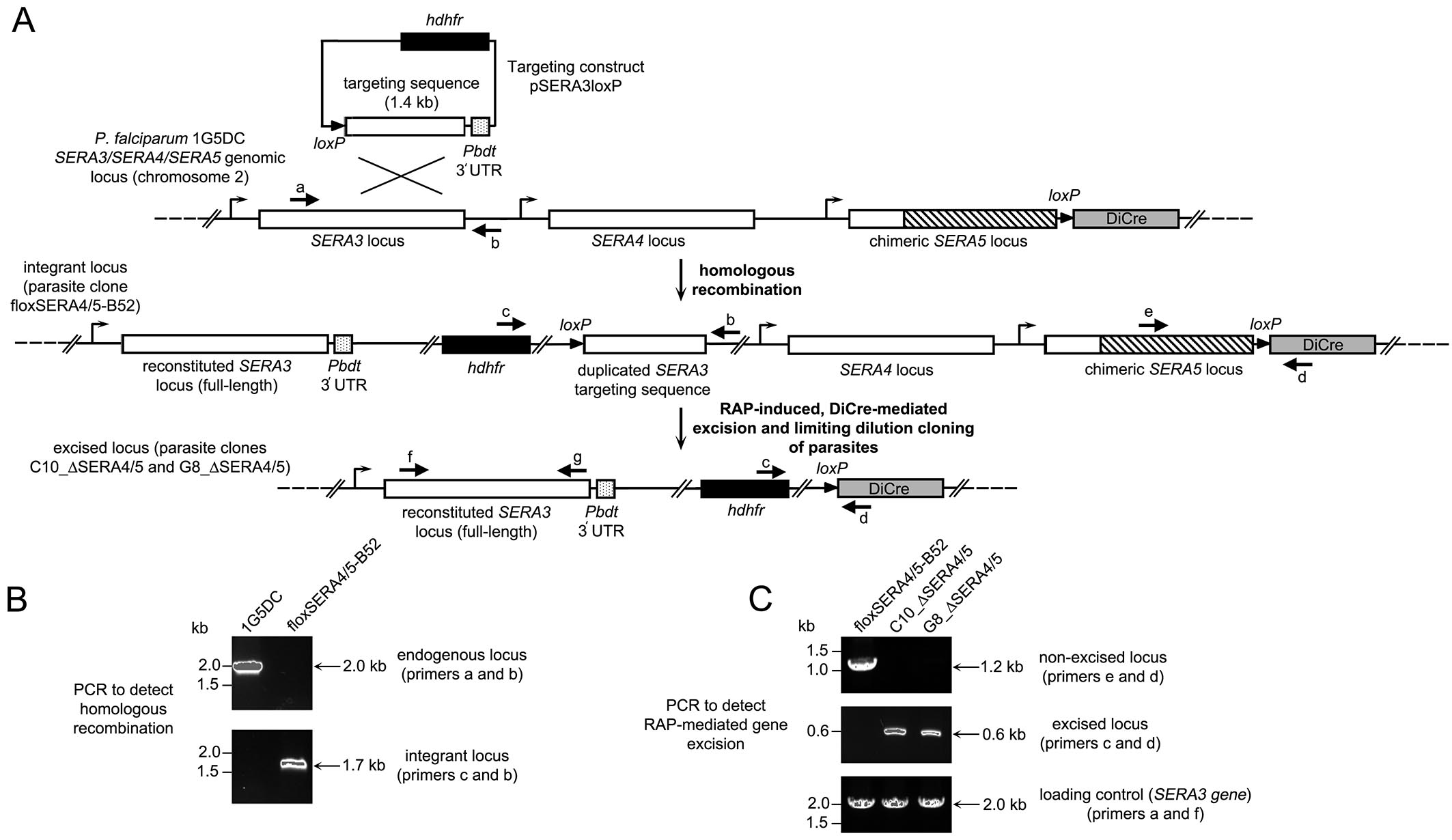

Supplement: S5 Fig — (A) Strategy for simultaneous conditional deletion of both the SERA4 and SERA5 genes. Targeting construct pSERA3loxP contains ~1.4 kb of 3´ SERA3 sequence to drive integration of the entire construct into the P. falciparum clone 1G5DC SERA3 locus by single-crossover homologous recombination. For clarity, the intron-exon structure of the SERA genes is not indicated. The targeting sequence extended to and included the SERA3 stop codon and was followed by the 3′ UTR of the P. berghei dihydrofolate reductase (Pbdt) gene to ensure correct regulation of the modified SERA3 gene. Correct integration was expected to reconstitute the gene whilst introducing a loxP site downstream of the introduced hdhfr selection cassette. DiCre-mediated recombination was predicted to excise the entire sequence between the loxP sites, including both the SERA4 and SERA5 genes. Positions of hybridisation of primers used for diagnostic PCR analysis of integration and excision events are shown as black arrows. Primer identities are: a, S3_F6; b, S3_DS_R1; c, CAM5´_R3; d, hsp86_3´_R1; e, sgS5_seq4F; f, S3_F7; g, S3_R1 (see S1 Table for sequences of all primers used in this study). (B) Diagnostic PCR analysis of genomic DNA from the parental 1G5DC P. falciparum clone and integrant clone floxSERA4/5-B52, confirming the predicted integration event. Expected sizes of the PCR amplicons are indicated. (C) PCR analysis of genomic DNA from RAP-treated or control parental integrant and two ΔSERA4/5 P. falciparum clones, confirming the predicted DiCre-mediated excision events. The expected sizes of the PCR amplicons specific for the intact, excised and control locus are indicated. (JPG) [file ppat.1006453.s005.jpg]

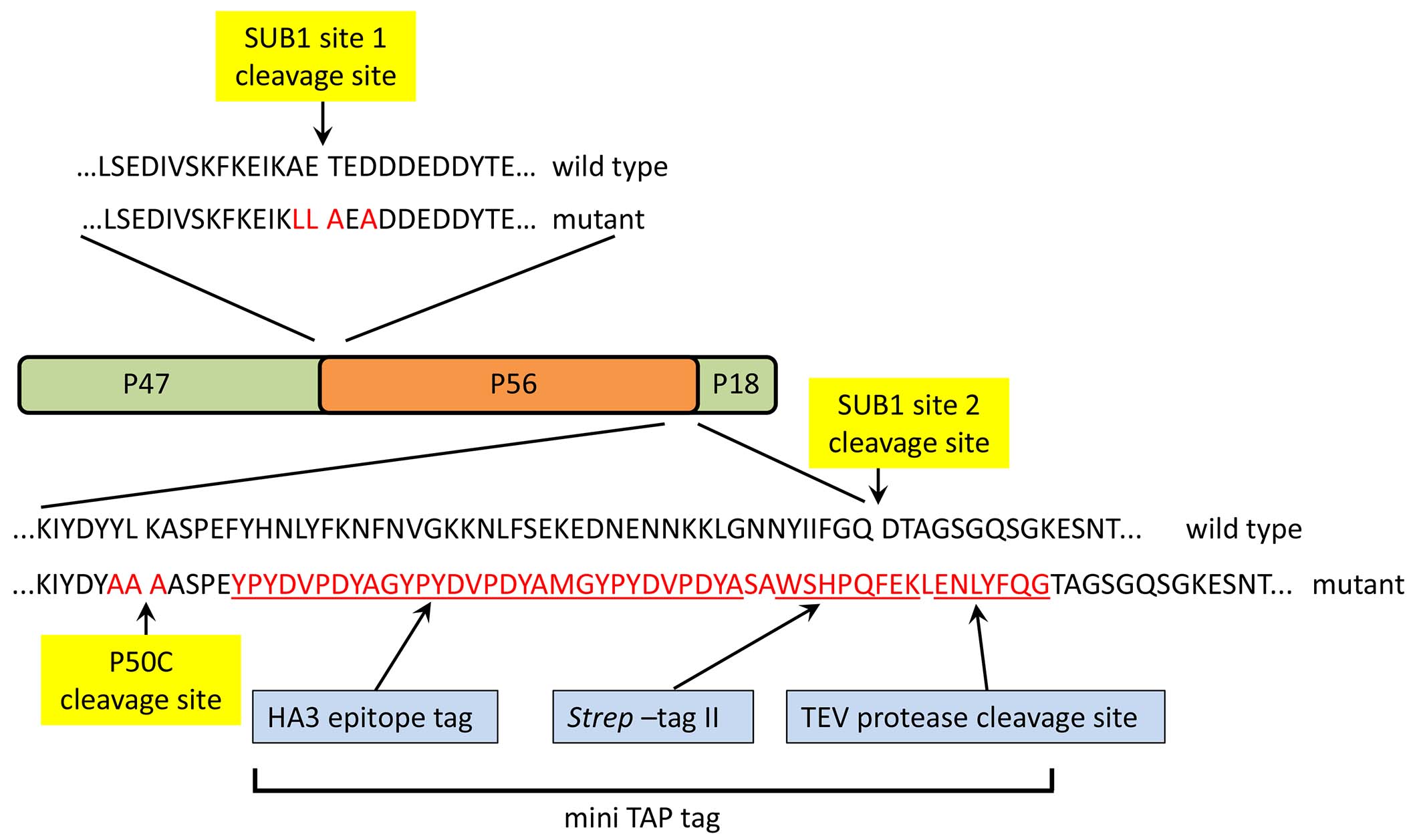

Supplement: S6 Fig — Schematic representation of the 3D7 P. falciparum SERA5 primary structure with the major processing fragments derived from SUB1 cleavage (P47, P56 and P18) indicated. The P56 fragment is trimmed near its C-terminus (to form the P50 terminal processing product; not depicted on this schematic) by further cleavage at the P50C cleavage site, mediated by an unknown cysteine protease called protease X [6]. The wild-type sequence flanking these sites is shown, alongside the modifications made (shown in red) by introduction of a mini TAP tag (major elements of which are also underlined and indicated) as well as introduction of several substitutions at the P50C and SUB1 site 1 cleavage sites. Note that insertion of the mini TAP tag sequence modifies the SUB1 site 2 cleavage site but has previously been shown to be tolerated when introduced into the endogenous SERA5 gene [6]. All the modifications shown in red were present in the mutant SERA5 gene expressed in plasmid construct pDC2_mC_sgS5mut, whereas only the mini TAP tag sequence was present in the pDC2_mC_sgS5 construct. (JPG) [file ppat.1006453.s006.jpg]
