# Supplementary material for: The Plasmodium falciparum pseudoprotease SERA5 regulates the kinetics and efficiency of malaria parasite egress from host erythrocytes
Source: PLoS Pathog. 2017 Jul 6;13(7):e1006453. doi: 10.1371/journal.ppat.1006453 (PMC5500368; doi:10.1371/journal.ppat.1006453)
Supplement: S1 Table — (PDF) [file ppat.1006453.s017.pdf]

S1 Table. Oligonucleotide primer sequences used in this study

**Primer name**	**sequence**
S4_F3	GAATTGTGCCTTATCATGGGCC
S4_HpaI_F	ATGAACAAAGGTTCAGTTAACGCTTATGTAAAAGCTG
S4_XhoI_R	ATATTTCATACTCGAGTTATACATAACAGAAATCAC
S4_F4	GAAGAACATAAAGAAGAAAACGATAATGTAC
S4_DS_R1	AATCTCATGAATAGCCTTCCATTTCG
CAM5'_R3	GTTTCTATAAATTGATATC
hsp86_3'_R1	GACTTTACTGAGACATG
sgS5_seq4F	CCGCGGATCATGCGGTGAACATTGTGGGC
S4_R2	GGGCCCTGTTTCTTGTTGTCCGC
S3_F1	GTTGAGGTTTACGTAGATATGTTTAAAGCAAATGAACATGG
S3_F2	GAAACATACGTAGCTGTGTCAGGAAGTGAAGTGAAGG
S3_R1	GATCCTTCTCGAGTTATATATAACAGAAAAAACAATC
S3_DS_R1	TTTTTCATGTATCCTTTC
S3_F6	GGTAGAAGCTTCTGACGAATGTTACAAG
S3_F7	GTGACGCAATAGCTTCAG
S5_US_F5	GTTTAAGTACgtATGATGACAAAAATGAATGTG
S5_US_R3	GACTTCcTTaaGATATATGAATATTAAG
sgS5_5'_F_AflII	CATATATCttaaggtttaagaggtaccatg
S5_st1II_R	CATCTTCATCATCAgcTTCGGcTaaCaaTTTG
S5_st1II_F	CAAAttGttAgCCGAAgcTGATGATGAAGATG
S5syn_F1	GTGGATATGTATGGCCCGACCCATTGCC
p50_F_New	GCAAGATCTACGATTATGCTGCGGCGGCGAGCCCGG
p50_R_New	CCGGGCTCGCCGCCGCAGCATAATCGTAGATCTTGC

* Restriction sites used in primers are underlined
